# Model-error estimation and model adaptivity for hyperbolic moment equations in one dimension

**DOI:** 10.1007/s10404-026-02912-0

**Published:** 2026-07-17

**Authors:** Rik Verbiest, Julian Koellermeier

**Affiliations:** 1 Bernoulli Institute, University of Groningen, Nijenborgh 9, Groningen, 9747 AG The Netherlands; 2 Department of Mathematics, Computer Science and Statistics, Ghent University, Krijgslaan 299 - S9, Ghent, 9000 Belgium

**Keywords:** Moment models, Multi-scale modelling and simulation, Nonequilibrium gas microflows, Rarefied gas dynamics

## Abstract

Microflows like Knudsen pumps often include rarefied gases featuring different degrees of rarefaction. This different modeling complexity requires space- and time-adaptive rarefied gas models that resolve the physical effects efficiently in each subdomain of the microflow. Different-order moment models are effective at describing rarefied gases in microflows with respective levels of complexity in each subdomain. In this work, analytical model-error estimators for the Hyperbolic Moment Equations (HME) model are derived that are then used to define a space- and time-adaptive moment model for microflows of rarefied gases. First, a domain decomposition strategy of the microflow into subdomains, each modelled by an HME model of an appropriate order, is presented, using domain decomposition criteria that are based on the exact model difference between a higher-order HME model and a lower-order HME model and on chosen error thresholds. Secondly, a non-linear adaptation of a recently developed padded buffer cell approach is presented to couple these varying-order HME models using a single finite volume scheme. Finally, a smoothing of the domain decomposition is proposed to limit oscillations generated by the coupling. While the performance depends on the thresholds and the smoothing parameter, the proposed adaptive moment model is able to capture the different degrees of rarefaction of the rarefied gas in the microflow and yields accurate numerical results compared to experimentally validated DVM benchmark data while obtaining computational speedups of up to 40 percent compared to using a high-order HME model in the entire domain.

## Introduction

Flows at microscopic scale often include rarefied gases that are characterized by the presence of both near-equilibrium regions and nonequilibrium regions. Examples are Knudsen layers in micro-scale gas flows (Zhang et al. 2006), Knudsen pumps (Wang et al. 2020), and micro electro mechanical systems (Ho and Tai 1998). This also extends to high-altitude flights (Ivanov and Gimelshein 1998) and atmospheric reentry flights (Li and Zhang 2009). In these examples, the dimensionless Knudsen number (Kn) varies in time and space. In regions of small Kn ($\text {Kn} \lesssim 0.01$), the gas can be assumed to be in equilibrium and standard fluid dynamics models for the macroscopic quantities yield sufficient accuracy. The nonequilibrium effects in regions of moderate Kn ($0.01 \lesssim \text {Kn} \lesssim 10$) to large Kn ($10 \lesssim \text {Kn}$) need models beyond the standard fluid equations that include microscopic effects to capture the gas dynamics (Struchtrup 2005). This motivates the need for adaptive models and hybrid methods for the numerical simulation of rarefied gas flows. 

Many hybrid methods couple fluid dynamics models such as the Euler equations or the Navier-Stokes equations in regions of small Kn with a particle representation in regions of moderate to large Kn (Garcia et al. 1999; Kolobov et al. 2005, 2007; Tiwari and Klar 1998; Wu et al. 2006; Schwartzentruber et al. 2007). The fluid dynamics equations are typically solved by *conventional continuum solvers* that discretize the continuum equations, while the particle description typically employs *particle-based* solvers that track sampled particles whose streaming and collision mimic the Boltzmann transport equation, such as the Direct Simulation Monte Carlo (DSMC) method (Bird 1963) that is used in Wu et al. (2006), Schwartzentruber et al. (2007), Garcia et al. (1999) as the particle solver, for example. The statistical nature of particle-based solvers poses challenges for the coupling of these solvers with the continuum solvers that simulate the continuum equations, due to the noise from the particle-based solver that enters the continuum solvers (Hash and Hassan 1997). Some authors propose kinetic schemes for continuum equations (Kolobov et al. 2005, 2007; Tiwari and Klar 1998) to avoid feeding noise to a continuum solver, but these methods result in additional computational cost compared to conventional continuum solvers.

Deterministic moment-closure models for kinetic equations, first introduced by Grad in Grad (1949), describe the evolution of macroscopic quantities by a finite set of moments approximating the distribution function. The resulting partial differential equations of the reduced order model are then discretized and numerically solved. They have proven to be a powerful modelling technique for the simulation of rarefied gases, both for general rarefied gases in the mesoscopic regime (Cai et al. 2015; Torrilhon 2016), including low-speed rarefied flows, where particle-based approaches such as DSMC may suffer from significant statistical noise and require extensive sampling to obtain smooth macroscopic quantities (Bird 1994), and micro-scale gas flows such as thermally driven microflows (Taheri and Struchtrup 2010), Knudsen pumps (Wang et al. 2020), and gas flows in slider bearings (Gu et al. 2016). Moment-closure models, such as the one-dimensional Hyperbolic Moment Equations (HME) (Cai et al. 2013) that are considered in this paper, achieve increasing accuracy by successively including more and more variables and evolution equations. A moment model is therefore characterized by its order $M\in \mathbb {N}$, measuring the number of variables or equations. A higher-order moment model of order $M_\textrm{H}\in \mathbb {N}$ is often more accurate but computationally more expensive than a lower-order moment model of order $M_\textrm{L}\in \mathbb {N}$, where $M_\textrm{H}>M_\textrm{L}$. Thus, it is desirable for computational efficiency to always use the moment model with the smallest possible order *M* that reaches acceptable accuracy. Moment models are ideally suited for adaptivity because they have a hierarchical structure such that the model error can be estimated directly and the same type of numerical solver can be used in the entire domain (Torrilhon and Sarna 2017).

Existing methods for model-adaptive simulation of moment models typically consist of two main steps: 1) domain decomposition into subdomains that are each modelled by a moment model of an appropriate order *M* and 2) spatial coupling of the subdomains that are each modelled by a different-order moment model. The domain decomposition in the first step is typically based on model-error estimators that estimate the model error of the currently used moment model. A proof of concept for a model-error estimation framework of hierarchical moment models has been proposed in Torrilhon and Sarna (2017). In Li et al. (2021), domain decomposition indicators for the three-dimensional HME model are constructed based on the moment realizability matrices proposed in Levermore et al. (1998). In contrast to the adaptive simulation method proposed in this paper, the lower-order HME model and the higher-order HME model are fixed in Li et al. (2021). The authors of Abdelmalik and Brummelen (2017) base their domain decomposition on a direct estimation of the model error by limiting the dimension of the moment approximation function space of a kinetic moment model. Heuristic model-error estimators based on (gradients of) the numerical solution are numerically studied for kinetic moment models in Koellermeier (2019). The authors of Schmeiser and Zwirchmayr (1998) use a heuristic procedure to vary the order of a linear kinetic moment model. The model-adaptive simulation of moment models has recently also gained interest in free-surface flow modelling. In Verbiest and Koellermeier (2026), the model error is estimated by computing the model difference between a higher-order moment model and a lower-order moment model exactly using the hierarchical structure of a shallow water moment model. For the spatial coupling in the second step, a padded buffer cell approach that adds artificial moments to compute the boundary interface fluctuations has recently been proposed and applied to a linear kinetic moment model (Verbiest and Koellermeier 2026) and a non-linear shallow water moment model (Verbiest and Koellermeier 2026).

In this paper, the first adaptive HME model that is adaptive in time and space is proposed and numerically simulated in one-dimensional space. The proof of concept introduced in this paper can then be extended in future research to higher-dimensional configurations for real-world applications. The adaptive procedure consists of a domain decomposition into subdomains based on analytical model-error estimators and a spatial coupling formula. The domain decomposition criterion is constructed by numerically computing the exact difference between a higher-order HME model and a lower-order HME model similar to what has been proposed in Verbiest and Koellermeier (2026) for shallow water moment models but extended to the HME model that is not strictly hierarchical. Moreover, the domain decomposition criterion is numerically evaluated at two discrete times using a predictor step with a computationally cheap numerical solver for robustness. The domain decomposition is smoothed to limit unphysical oscillations created at the boundary interfaces between the subdomains. The spatial coupling formula is a modification of the path-conservative padded buffer cell approach used in Verbiest and Koellermeier (2026). In particular, this allows to use the same type of numerical solver in the entire domain, which simplifies the coupling.

The remainder of the paper is organized as follows. In Sect. 2, the HME model is briefly reviewed. The domain decomposition criteria and the coupling of the subdomains are discussed in Sect. 3. In Sect. 4, numerical simulations are performed to test the adaptive procedure by means of two test cases. While the first test case is a proof of concept to analyze the performance of the domain decomposition criteria, the second test case is a shock tube for which the results are compared with a benchmark DVM method, which has been validated with experimental data in the literature (Mieussens 2000). The paper ends with a brief conclusion.

## Hyperbolic Moment Equations (HME)

In this paper, we derive efficient numerical solutions for the one-dimensional Boltzmann transport equation (BTE) with Bhatnagar-Gross-Krook (BGK) collision operator (Bhatnagar et al. 1954)

1 $$\begin{aligned}&\partial _t f(t,x,c) + c\, \partial _x f(t,x,c) \nonumber \\& \quad = -\frac{1}{\tau }\left( f(t,x,c) - f_\mathrm{{Maxw}}^{[\rho ,u,\theta ]}(t,x,c)\right) \end{aligned}$$

governing the evolution of the particle distribution function $f(t,x,c):\mathbb {R}^+\times \Omega _x\times \Omega _c\mapsto \mathbb {R}^+$ that describes the probability of finding a gas particle at time $t\in \mathbb {R}^+$ in position $x\in \Omega _x\subset \mathbb {R}$ and with particle velocity $c\in \Omega _c\subset \mathbb {R}$. The right-hand side BGK term models the relaxation of *f(t,x,c)* with relaxation time $\tau \in \mathbb {R}^+$ towards a Maxwellian distribution

2 $$\begin{aligned} f_\mathrm{{Maxw}}^{[\rho ,u,\theta ]}(t,x,c)=\frac{\rho (t,x)}{\sqrt{2\pi \theta (t,x)}}\exp \left( -\frac{(c-u(t,x))^2}{2\theta (t,x)}\right) , \end{aligned}$$

with density $\rho (t,x):\mathbb {R}^+\times \Omega _x\mapsto \mathbb {R}^+$, velocity $u(t,x):\mathbb {R}^+\times \Omega _x\mapsto \mathbb {R}$, and temperature $\theta (t,x):\mathbb {R}^+\times \Omega _x\times \mapsto \mathbb {R}^+$, that are defined via the moments of *f(t,x,c)*:

3 $$\begin{aligned} \rho (t,x) & = \int _{\Omega _c} f(t,x,c)dc, \\&\quad\quad\quad \rho (t,x)u(t,x)\nonumber \\& = \int _{\Omega _c} c f(t,x,c)dc, \end{aligned}$$

4 $$\begin{aligned}&\frac{1}{2}\rho (t,x)\left( u(t,x)^2+\theta (t,x)\right) \nonumber \\& \quad =\int _{\Omega _c} c^2 f(t,x,c)dc. \end{aligned}$$

The one-dimensional Hyperbolic Moment Equations (HME), proposed in Cai et al. (2013), are derived by assuming *f(t,x,c)* to be a truncated Hermite expansion of order *M\ge 2* given by

5 $$\begin{aligned}&f(t,x,c) = \sum _{i=0}^{M} f_i(t,x)\frac{1}{\sqrt{2\pi }}\theta (t,x)^{-\frac{i+1}{2}}\nonumber \\& \quad \textrm{He}_i\left( \frac{c-u(t,x)}{\sqrt{\theta (t,x)}}\right) \exp \left( -\frac{(c-u(t,x))^2}{2\theta (t,x)}\right) . \end{aligned}$$

Here, $\textrm{He}_i(\zeta ):\mathbb {R}\mapsto \mathbb {R}$ and $f_i(t,x):\mathbb {R}^+\times \Omega _x\mapsto \mathbb {R}$ are the *i-*th Hermite polynomial and moment, respectively. For compatibility with the definitions of *ρ (t,x)*, *u(t,x)* and *θ (t,x)* in Eqs. (3), (4), the first three moments read $f_0(t,x)\equiv \rho (t,x)$ and $f_1(t,x)\equiv f_2(t,x)\equiv 0$. For notational simplicity, we suppress the explicit dependence on (*t*, *x*) in the remainder of this paper for all functions whenever no confusion arises. The density *ρ*, velocity *u*, and temperature *θ* are governed by the equations

6 $$\begin{aligned} \partial _t \rho + u \partial _x \rho + \rho \partial _x u&= 0, \end{aligned}$$

7 $$\begin{aligned} \partial _t u + \frac{\theta }{\rho } \partial _x \rho + u\partial _x u +\partial _x\theta&= 0, \end{aligned}$$

8 $$\begin{aligned} \partial _t \theta + 2\theta \partial _x u + u\partial _x \theta +\frac{6}{\rho }\partial _x f_3&= 0. \end{aligned}$$

Equations for the moments $f_k$, *k= 3,… ,M*, are obtained in Cai et al. (2013) by taking higher-order moments of the BTE (1)

9 $$\begin{aligned} \begin{aligned}&\partial _t f_k - f_{k-1}\frac{\theta }{\rho }\partial _x \rho +(k+1)f_k\partial _x u \\& \quad +\left( \frac{1}{2}\theta f_{k-3}+\frac{k-1}{2}f_{k-1} \right) \partial _x \theta \\& \quad -\frac{3}{\rho }f_{k-2}\partial _x f_3+\theta \partial _x f_{k-1}+u\partial _x f_k \\& \quad +(k+1)\partial _x f_{k+1} = -\frac{1}{\tau }f_k. \end{aligned} \end{aligned}$$

The equations for $f_k$, *k= 3,… ,M-1*, in the HME model are given by the respective Eq. (9). The equation for the highest-order moment $f_M$ is closed by setting $f_{M+1}=0$ (which follows from taking the respective moment of the ansatz (5)) and adding a hyperbolic regularization term $\mathcal {R}_M\in \mathbb {R}$ given by

10 $$\begin{aligned} \mathcal {R}_M=\frac{M+1}{2}\left( 2f_M\partial _x u +f_{M-1}\partial _x\theta \right) \end{aligned}$$

to the left-hand side of the respective Eq. (9). The equation for the highest-order moment $f_M$ then becomes

11 $$\begin{aligned} \begin{aligned}&\partial _t f_M - f_{M-1}\frac{\theta }{\rho }\partial _x \rho +\left( \frac{1}{2}\theta f_{M-3}+f_{M-1} \right) \partial _x \theta \\& \quad - \frac{3}{\rho }f_{M-2}\partial _x f_3+\theta \partial _x f_{M-1}+u\partial _x f_M \\& \quad = -\frac{1}{\tau }f_M.\end{aligned} \end{aligned}$$

The hyperbolic regularization (10) overcomes the loss of hyperbolicity of the original Grad moment system and can be seen as a particular closure. For details, we refer the reader to Cai et al. (2013). The three Eqs. (6), (7), (8) together with the moment Eq. (9) (for $f_k$, *k= 3,… ,M-1*) and (11) (for $f_M$) define the closed HME model of order *M*. The HME model of order *M* can be written in compact form as

12 $$\begin{aligned} \partial _t \vec {w}_M + A_M(\vec {w}_M) \partial _x \vec {w}_M = S_M \vec {w}_M, \end{aligned}$$

with state variable vector $\vec {w}_M=\left( \rho ,u,\theta ,f_3,\ldots ,f_M\right) \in \mathbb {R}^{M+1}$, non-linear transport matrix $A_M(\vec {w}_M)\in \mathbb {R}^{(M+1)\times (M+1)}$ given by

13 $$\begin{aligned} & A_M(\vec {w}_M)\nonumber \\ & =\begin{pmatrix} u & \rho & & & & & & \\ \frac{\theta }{\rho }& u & 1 & & & & & \\ & 2\theta & u & \frac{6}{\rho } & & & & \\ & 4f_3 & \frac{\rho \theta }{2} & u & 4 & & & \\ -\frac{\theta f_3}{\rho } & 5f_4 & \frac{3f_3}{2} & \theta & u & 5 & & \\ \vdots & \vdots & \vdots & \vdots & \ddots & \ddots & \ddots & \\ -\frac{\theta f_{M-2}}{\rho }& Mf_{M-1} & \frac{(M-2)f_{M-2}+\theta f_{M-4}}{2} & -\frac{3f_{M-3}}{\rho } & & \theta & u & M \\ -\frac{\theta f_{M-1}}{\rho }& & f_{M-1}+\frac{\theta f_{M-3}}{2} & -\frac{3f_{M-2}}{\rho } & & & \theta & u \end{pmatrix} , \end{aligned}$$

and the constant collision matrix $S_M\in \mathbb {R}^{(M+1)\times (M+1)}$ given by

14 $$\begin{aligned} S_M=-\frac{1}{\tau }\textrm{diag}(0,0,0,1,\ldots ,1). \end{aligned}$$

Note that from now on we denote an HME model of a certain order *M* by the short notation $\text {HME}_{M}$.

## Adaptive simulation of HME

In this section, an algorithm for the model-adaptive simulation of the HME (12) is presented. The algorithm is based on the recently proposed algorithm for the adaptive simulation of hierarchical moment models for shallow free surface flows (Verbiest and Koellermeier 2026) and consists of two steps: (1) domain decomposition into subdomains that are each modelled by a moment model of an appropriate order *M* and (2) spatial coupling of the subdomains that are each modelled by a different-order moment model.

The domain decomposition in the first step is based on model-error estimators that estimate the error that is made by restricting the Hermite expansion (5) to the first *M+1* moments. The idea is similar to the framework proposed in Torrilhon and Sarna (2017). The model-error estimator is then used to construct a set of domain decomposition criteria. These domain decomposition criteria are evaluated on the grid, effectively decomposing the physical domain $\Omega _x$ into subdomains that are each modelled by an HME model of an appropriate order *M*. Although the HME have a hierarchical structure, lower-order HME models are not necessarily special cases of higher-order HME models, because of the hyperbolic regularization term $\mathcal {R}_M$ (10) that is added to the equation for the highest order moment. As a result, the algorithm proposed in Verbiest and Koellermeier (2026) needs to be adjusted. We propose a numerical approximation of the spatial derivatives appearing in the exact difference between a higher-order $\text {HME}_{M_\textrm{H}}$ model and a lower-order $\text {HME}_{M_\textrm{H}-2}$ model, where $M_\textrm{H}\ge 4$, both at the current time step and at the next time step, where the discrete values at the next time step are predicted using a computationally cheap finite volume method. This is discussed in Sect. 3.1 and in Sect. 3.3.

For the spatial coupling of the different-order subdomains at their boundary interfaces in the second step, we use an adaptation of the path-conservative padded buffer cell method proposed in Verbiest and Koellermeier (2026) that adds additional moments equal to zero to match the number of variables at the boundary interface. In the interior of each subdomain, the numerical scheme employs a proper characteristic decomposition, whereas at the boundary interfaces, the numerical scheme does not use a characteristic decomposition and uses additional numerical viscosity to limit unphysical oscillations caused by the artificial jump in the higher-order moments. This is discussed in Sect. 3.2.

The domain decomposition in the first step assigns an order *M* to each cell of the discretized domain. The spatial coupling in the second step creates small unphysical oscillations at the boundary interfaces. To avoid that the unphysical oscillations interact with each other, a smoothing of the domain decomposition is proposed in Sect. 3.4.

### Remark 1

It has been observed that the numerical simulations obtained using the HME model show a decoupling for even and odd *M*, see Fan and Koellermeier (2020) and Koellermeier and Torrilhon (2017), for example. This is due to the different characteristic speeds of the respective models, because they are tied to the roots of the Hermite polynomial $\textrm{He}_{M+1}$, whose structure differs between even and odd orders. Therefore, we will only consider even orders $M\in \{m\in \mathbb {N}\,|\,m\text { is even and }m>0\}$ in this paper.

### Remark 2

We construct domain decomposition criteria for switching between a higher-order $\text {HME}_{M_\textrm{H}}$ and a lower-order $\text {HME}_{M_\textrm{L}}$, where $M_\textrm{H}>M_\textrm{L}\ge 2$. In this paper, we only consider an increase or a reduction of two orders to allow for an efficient implementation, i.e., $M_\textrm{L}=M_\textrm{H}-2$, with $M_\textrm{H}\ge 4$, similar to Verbiest and Koellermeier (2026). Similar ideas to those discussed in this section could be applied to allow for arbitrary (even) order increases or reductions.

### Model-error estimation

The domain decomposition criteria proposed in this paper are based on a model-error estimator that estimates the model difference between a higher-order $\text {HME}_{M_\textrm{H}}$ and a lower-order $\text {HME}_{M_\textrm{H}-2}$ directly. Consider a higher-order $\text {HME}_{M_\textrm{H}}$ and a lower-order $\text {HME}_{M_\textrm{H}-2}$. A direct comparison of the first $M_\textrm{H}-1$ Eq. (9) of the $\text {HME}_{M_\textrm{H}}$ with the Eq. (9) of the $\text {HME}_{M_\textrm{H}-2}$ shows that the $\text {HME}_{M_\textrm{H}}$ adds an additional term $\Delta _{M_\textrm{H},M_\textrm{H}-2}\in \mathbb {R}$, the model difference, to the equation for the $(M_\textrm{H}-2)$th moment $f_{M_\textrm{H}-2}$, where

15 $$\begin{aligned}&\Delta _{M_\textrm{H},M_\textrm{H}-2}=(M_\textrm{H}-1)\partial _x f_{M_\textrm{H}-1}\nonumber \\& \quad + \frac{M_\textrm{H}-1}{2}\left( 2f_{M_\textrm{H}-2}\partial _x u +f_{M_\textrm{H}-3}\partial _x\theta \right) . \end{aligned}$$

The first term on the right-hand side of (15) originates directly from the moment Eq. (9) for the higher-order $\text {HME}_{M_\textrm{H}}$, whereas the second term on the right-hand side of (15) is a result of the hyperbolic regularization term (10) which is present in the lower-order $\text {HME}_{M_\textrm{H}-2}$. The model-error estimators that are used in the model-refinement criteria (i.e., increasing the order *M* of the HME model) and in the model-coarsening criteria (i.e., decreasing the order *M* of the HME model) are based on estimating the model difference $\Delta _{M_\textrm{H},M_\textrm{H}-2}$ (15).

#### Model-refinement criterion

Assume that the rarefied gas is modelled by the lower-order $\text {HME}_{M_\textrm{H}-2}$. The term containing $f_{M_\textrm{H}-1}$ in the expression for the model difference $\Delta _{M_\textrm{H},M_\textrm{H}-2}$ (15) is not defined in the lower-order $\text {HME}_{M_\textrm{H}-2}$ and needs a special treatment. We propose two model-error estimators for model-refinement $\mathcal {E}^{(1)}_{M_\textrm{H}-2\rightarrow M_\textrm{H}}\in \mathbb {R}$ and $\mathcal {E}^{(2)}_{M_\textrm{H}-2\rightarrow M_\textrm{H}}\in \mathbb {R}$, based on the exact model difference $\Delta _{M_\textrm{H},M_\textrm{H}-2}$ (15) and with a heuristic treatment for the undefined terms:

16 $$\begin{aligned} \mathcal {E}^{(1)}_{M_\textrm{H}-2\rightarrow M_\textrm{H}}\, {:}{=} \,&\frac{M_\textrm{H}-1}{2}\left( 2f_{M_\textrm{H}-2}\partial _x u +f_{M_\textrm{H}-3}\partial _x\theta \right) , \end{aligned}$$

17 $$\begin{aligned}&\mathcal {E}^{(2)}_{M_\textrm{H}-2\rightarrow M_\textrm{H}}\,{:}{=} \,(M_\textrm{H}-1)\partial _x f^{\textrm{approx}}_{M_\textrm{H}-1}\nonumber \\& \quad + \frac{M_\textrm{H}-1}{2}\left( 2f_{M_\textrm{H}-2}\partial _x u +f_{M_\textrm{H}-3}\partial _x\theta \right) , \end{aligned}$$

where $f^{\textrm{approx}}_{M_\textrm{H}-1}$ is an approximation of the true $f_{M_\textrm{H}-1}$ that is not included in the $\text {HME}_{M_\textrm{H}-2}$. The estimator $\mathcal {E}^{(1)}_{M_\textrm{H}-2\rightarrow M_\textrm{H}}$ (16) neglects the contribution of the term that is not defined in the $\text {HME}_{M_\textrm{H}-2}$, whereas the estimator $\mathcal {E}^{(2)}_{M_\textrm{H}-2\rightarrow M_\textrm{H}}$ (17) approximates this contribution by an approximate $f^{\textrm{approx}}_{M_\textrm{H}-1}$. Note that $\mathcal {E}^{(1)}_{M_\textrm{H}-2\rightarrow M_\textrm{H}}$ (16) and $\mathcal {E}^{(2)}_{M_\textrm{H}-2\rightarrow M_\textrm{H}}$ (17) coincide for the approximation $f^{\textrm{approx}}_{M_\textrm{H}-1}\equiv 0$. The model-refinement criterion is defined in Definition 1.

##### Definition 1

*(Model-refinement criterion)* Let $M_\textrm{H}\ge 4$. The model-refinement criterion $\textrm{R}_{M_\textrm{H}-2\rightarrow M_\textrm{H}}\in \{0,1\}$ is defined as

18 $$\begin{aligned} \textrm{R}_{M_\textrm{H}-2\rightarrow M_\textrm{H}} \, {:}{=}\, \textbf{1}\!\left[ \max \left( \left| \mathcal {E}^{(1)}_{M_\textrm{H}-2\rightarrow M_\textrm{H}}\right| ,\left| \mathcal {E}^{(2)}_{M_\textrm{H}-2\rightarrow M_\textrm{H}}\right| \right)>\epsilon _\textrm{R}\right] , \end{aligned}$$

where $\mathcal {E}^{(1)}_{M_\textrm{H}-2\rightarrow M_\textrm{H}}$ and $\mathcal {E}^{(2)}_{M_\textrm{H}-2\rightarrow M_\textrm{H}}$ are defined by (16) and (17), respectively, where $\epsilon _\textrm{R}>0$ is a chosen refinement threshold, and where $\textbf{1}\!\left[ \cdot \right]$ is the indicator function that is defined as $\textbf{1}\!\left[ A\right] =1$ if the statement *A* holds and $\textbf{1}\!\left[ A\right] =0$ otherwise. $\textrm{R}_{M_\textrm{H}-2\rightarrow M_\textrm{H}}=1$ signals model refinement.

Considering two model-error estimators for model refinement $\mathcal {E}^{(1)}_{M_\textrm{H}-2\rightarrow M_\textrm{H}}$ (16) and $\mathcal {E}^{(2)}_{M_\textrm{H}-2\rightarrow M_\textrm{H}}$ (17) is motivated by their robust discretization, discussed in Sect. 3.3. For numerical simulations, the model-error estimators for model refinement $\mathcal {E}^{(1)}_{M_\textrm{H}-2\rightarrow M_\textrm{H}}$ (16) and $\mathcal {E}^{(2)}_{M_\textrm{H}-2\rightarrow M_\textrm{H}}$ (17) need to be discretized and used as the basis for a discretization of the model-refinement criterion $\textrm{R}_{M_\textrm{H}-2\rightarrow M_\textrm{H}}$ (18). In Sect. 3.3, discretizations for both estimators $\mathcal {E}^{(1)}_{M_\textrm{H}-2\rightarrow M_\textrm{H}}$ (16) and $\mathcal {E}^{(2)}_{M_\textrm{H}-2\rightarrow M_\textrm{H}}$ (17) will be proposed that follow naturally from computing the model-difference (15) at two different discrete times. To be more precise, $\mathcal {E}^{(1)}_{M_\textrm{H}-2\rightarrow M_\textrm{H}}$ (16) will be discretized using the already computed discrete values at a certain discrete time, because the higher-order moment $f_{M_{\textrm{H}}-1}$ equals zero at that time. $\mathcal {E}^{(2)}_{M_\textrm{H}-2\rightarrow M_\textrm{H}}$ (17) will be computed using predicted state variables, including a prediction for the undefined moment $f_{M_{\textrm{H}}-1}$ that will be used as $f_{M_{\textrm{H}}-1}^{\text {approx}}$ in computing $\mathcal {E}^{(2)}_{M_\textrm{H}-2\rightarrow M_\textrm{H}}$ (17), at the subsequent discrete time.

#### Model-coarsening criterion

Assume now that the rarefied gas is modelled by the higher-order $\text {HME}_{M_\textrm{H}}$. Since, in this case, all terms in expression (11) are defined, the model error estimator for model coarsening $\mathcal {E}_{M_\textrm{H}\rightarrow M_\textrm{H}-2}\in \mathbb {R}$ is simply given by the model difference $\Delta _{M_\textrm{H},M_\textrm{H}-2}$ (15):

19 $$\begin{aligned}&\mathcal {E}_{M_\textrm{H}\rightarrow M_\textrm{H}-2} \,{:}{=} \, (M_\textrm{H}-1)\partial _x f_{M_\textrm{H}-1}\nonumber \\& \quad + \frac{M_\textrm{H}-1}{2}\left( 2f_{M_\textrm{H}-2}\partial _x u +f_{M_\textrm{H}-3}\partial _x\theta \right) . \end{aligned}$$

We then define the model-coarsening criterion $\textrm{C}_{M_\textrm{H}\rightarrow M_\textrm{H}-2}$:

##### Definition 2

*(Model-coarsening criterion)* Assume that $M_\textrm{H}\ge 4$. The model-coarsening criterion $\textrm{C}_{M_\textrm{H}\rightarrow M_\textrm{H}-2} \in \{0,1\}$ is defined as

20 $$\begin{aligned} \textrm{C}_{M_\textrm{H}\rightarrow M_\textrm{H}-2} \,{:}{=}\, \textbf{1}\!\left[ \left| \mathcal {E}_{M_\textrm{H}\rightarrow M_\textrm{H}-2}\right| < \epsilon _\textrm{C}\right] , \end{aligned}$$

where $\mathcal {E}_{M_\textrm{H}\rightarrow M_\textrm{H}-2}$ is defined by (19), where $\epsilon _\textrm{C}>0$ is a chosen coarsening threshold, and where $\textbf{1}\!\left[ \cdot \right]$ is the indicator function that is defined as $\textbf{1}\!\left[ A\right] =1$ if the statement *A* holds and $\textbf{1}\!\left[ A\right] =0$ otherwise. $\textrm{C}_{M_\textrm{H}\rightarrow M_\textrm{H}-2}=1$ signals model coarsening.

The model-error estimator for model coarsening (19) is numerically evaluated and used as the basis for a discretization of the model-coarsening criterion $\textrm{C}_{M_\textrm{H}\rightarrow M_\textrm{H}-2}$ (20) in Sect. 3.3.

##### Remark 3

The authors of Tiwari and Klar (1998) decompose a 3D gas domain into continuum domains in which the Euler equations are simulated and into kinetic regimes in which the BTE (1) is simulated by computing the deviatoric stress tensor and the heat flux. These quantities are moments of the distribution function that vanish when the distribution function is a Maxwellian. In Kolobov et al. (2005, 2007), a heuristic domain decomposition criterion is used that is based on gradients of the density, velocity, pressure, and temperature. The domain decomposition criteria (18) and (20) are based on the model-error estimators (16)-(17) and (19). These estimators naturally include the higher-order moments and the spatial gradients of the velocity and the temperature by computing the exact model difference between a higher-order $\text {HME}_{M_\textrm{H}}$ and a lower-order $\text {HME}_{M_\textrm{H}-2}$. Different from the approaches in the literature, the error estimators (16)-(17) and (19) can be used for different orders $M_\textrm{H}$ and are not bound to a specific model choice like the ones from the literature discussed above. It is interesting to note that the density gradient does not appear explicitly in the model-error estimators (16)-(17) and (19), but is used in the prediction of the state variables at the next time step. This will be discussed in Sect. 3.3.

### Spatial coupling using padded buffer cell

After a suitable domain decomposition following Sect. 3.1 that decomposes the domain into subdomains that are each modelled by an HME moment model of an appropriate order *M*, the different-order HME models need to be coupled at their boundary interfaces. One advantage of the adaptive procedure proposed in this paper is that the same type of numerical method can be used in the entire domain with only minor adjustments at the boundary interfaces that do not cause significant computational overhead. The remainder of this section is organized as follows. First, the spatial discretization notation and the global finite volume discretization are discussed in Sects. 3.2.1 and 3.2.2, respectively. Next, the spatial coupling of the different-order subdomains based on the path-conservative padded buffer cell approach proposed in Verbiest and Koellermeier (2026) is presented in Sect. 3.2.3. Then, the numerical evaluation of the model-error estimators (16)-(17) and (19) and the domain decomposition criteria (18) and (20) is discussed in Sect. 3.3. Finally, the adaptive algorithm with smoothed domain decomposition is summarized in Sect. 3.4.

#### Spatial discretization notation

Equation (12) is discretized on a domain $\Omega _x$ with grid size *Δ x*

and discretized in time with time step *Δ t*, yielding the discretized cell-averaged moment vectors

21 $$\begin{aligned} w_i^n=\left( \rho _i^n,u_i^n,\theta _i^n,(f_3)_i^n,\ldots ,(f_M)_i^n\right) ^T \in \mathbb {R}^{M+1} \end{aligned}$$

in grid cells $\mathcal {C}_i=[x_i-\Delta x/2,x_i+\Delta x/2]$ with equidistant cell centers $x_i$, $i=1,2,\ldots ,N_x$, and at discrete times $t_n$, $n=0,1,\ldots ,N_t$. Note that the vector notation is dropped for simplicity and to distinguish the discretized moment vectors from the unknown functions described by Eq. (12). In particular, the subscript *i* in $w_i$ denotes the cell-averaged values in the cell labeled *i* and should not be confused with the subscript *M* in $\vec {w}_M$ in Eq. (12) that denotes the order of the moment model.

#### Global finite volume discretization

The overarching finite volume solver for the numerical simulation of the HME Eq. (12) utilizes a first-order time-splitting scheme. Equation (12) is split into a transport part and a collision part, yielding the two subsequent subproblems

22 $$\begin{aligned}&\partial _t \vec {w}_M + A_M(\vec {w}_M)\partial _x\vec {w}_M = 0, \end{aligned}$$

23 $$\begin{aligned}&\partial _t \vec {w}_M \phantom{\;\, + A_M(\vec {w}_M)\partial _x\vec {w}_M}= S_M\vec {w}_M. \end{aligned}$$

For the numerical simulation of the transport step (22), we consider a finite volume scheme and choose the Polynomial Viscosity Method (PVM) (Castro Díaz and Fernández-Nieto 2012) notation, because it is generally applicable to non-conservative systems including the HME model (12) and it includes well-known schemes such as the method of Osher and Solomon (Osher and Solomon 1982) extended to non-conservative systems (Dumbser and Toro 2011) and the PRICE scheme (Canestrelli et al. 2009), which will be used for the numerical simulations in Sect. 4. Note that a non-conservative formulation is necessary, since the highest-order moment Eq. (11) cannot be written in conservative form because of the hyperbolic regularization term (10) it contains. The PVM for the transport step (22) of the $\text {HME}_M$ (12), with a global order *M*, reads

24 $$\begin{aligned} w_i^{n+1,*} = w_i^n - \frac{\Delta t}{\Delta x}\left( D_{i-\frac{1}{2}}^+ + D_{i+\frac{1}{2}}^- \right) , \qquad i=1,\ldots ,N_x. \end{aligned}$$

The quantities $D_{i+\frac{1}{2}}^\pm =A_{\Phi }^\pm (w_i^n,w_{i+1}^n)\in \mathbb {R}^{M+1}$ are the one-sided interface fluctuations, i.e., the right- and left-going contributions of the jump between neighboring cell averages. They are defined by

25 $$\begin{aligned} A_{\Phi }^{\pm }(w_\mathrm{{l}},w_\mathrm{{r}}) = \frac{1}{2}\left( A_{\Phi }(w_\mathrm{{l}},w_\mathrm{{r}}) (w_\mathrm{{r}}-w_\mathrm{{l}}) \pm Q_{\Phi }(w_\mathrm{{l}},w_\mathrm{{r}}) (w_\mathrm{{r}}-w_\mathrm{{l}}) \right) , \end{aligned}$$

where the generalized Roe linearization $A_{\Phi }(w_\mathrm{{l}},w_\mathrm{{r}})\in \mathbb {R}^{(M+1)\times (M+1)}$ is computed via

26 $$\begin{aligned} A_{\Phi }(w_\mathrm{{l}},w_\mathrm{{r}}) (w_\mathrm{{r}}-w_\mathrm{{l}})=\int _{0}^1 A_M(\Phi (s;w_\mathrm{{l}},w_\mathrm{{r}}))\partial _s \Phi (s;w_\mathrm{{l}},w_\mathrm{{r}})ds \end{aligned}$$

and represents the interface contribution of the transport matrix $A_M(\vec {w}_M)$. Here,

27 $$\begin{aligned} \Phi (s;w_\mathrm{{l}},w_\mathrm{{r}}):[0,1]\mapsto \mathbb {R}^{M+1} \end{aligned}$$

denotes a path connecting the left state $w_\mathrm{{l}}\in \mathbb {R}^{M+1}$ and the right state $w_\mathrm{{r}}\in \mathbb {R}^{M+1}$ at the cell interface, such that $\Phi (0;w_\mathrm{{l}},w_\mathrm{{r}})=w_\mathrm{{l}}$ and $\Phi (1;w_\mathrm{{l}},w_\mathrm{{r}})=w_\mathrm{{r}}$. In this paper, the linear path

28 $$\begin{aligned} \Phi _{\text {lin}}(s;w_\mathrm{{l}},w_\mathrm{{r}})=(1-s) w_\mathrm{{l}} + s w_\mathrm{{r}} \end{aligned}$$

is used, which simplifies the generalized Roe linearization $A_{\Phi _{\text {lin}}}(w_\mathrm{{l}},w_\mathrm{{r}})$ (26) to a single integral of $A_M(\Phi _\textrm{lin}(s;w_\mathrm{{l}},w_\mathrm{{r}}))$ along the path

29 $$\begin{aligned} A_{\Phi _\textrm{lin}}(w_\mathrm{{l}},w_\mathrm{{r}})=\int _{0}^1 A_M(\Phi _\textrm{lin}(s;w_\mathrm{{l}},w_\mathrm{{r}}))ds. \end{aligned}$$

The integral in the definition of the generalized Roe linearization (29) needs to be approximated numerically. It was observed in Koellermeier (2017) that the Gauss-Legendre quadrature rule with three quadrature nodes yields sufficient accuracy for the HME. Moreover, the influence of the choice of the path $\Phi (s;w_\mathrm{{l}},w_\mathrm{{r}})$ on the numerical solution vanishes in many application cases provided the quadrature rule is chosen accurate enough (Rhebergen et al. 2008), as observed for the HME model in Koellermeier and Torrilhon (2017), for example.

The numerical viscosity matrix

30 $$\begin{aligned} Q_{\Phi _{\text {lin}}}(w_\mathrm{{l}},w_\mathrm{{r}})=P(A_{\Phi _{\text {lin}}}(w_\mathrm{{l}},w_\mathrm{{r}}))\in \mathbb {R}^{(M+1)\times (M+1)} \end{aligned}$$

is a function *P(· )* of the generalized Roe linearization and controls the amount of dissipation introduced at the interface.

For the linear path $\Phi _{\text {lin}}(s;w_\mathrm{{l}},w_\mathrm{{r}})$, the method of Osher and Solomon (Dumbser and Toro 2011; Osher and Solomon 1982) uses the numerical viscosity matrix

31 $$\begin{aligned} Q_{\Phi _{\text {lin}}}^\mathrm{{OS}}(w_\mathrm{{l}},w_\mathrm{{r}})=\int _{0}^1 \left| A_M(\Phi _{\text {lin}}(s;w_\mathrm{{l}},w_\mathrm{{r}}))\right| ds, \end{aligned}$$

and the PRICE scheme (Canestrelli et al. 2009) uses the numerical viscosity matrix

32 $$\begin{aligned} Q_{\Phi _{\text {lin}}}^\mathrm{{PRICE}}(w_\mathrm{{l}},w_\mathrm{{r}}) = \frac{\Delta x}{2\Delta t}I+\frac{\Delta t}{2\Delta x}A^2_{\Phi _{\text {lin}}}(w_\mathrm{{l}},w_\mathrm{{r}}). \end{aligned}$$

The method of Osher and Solomon (31) is an upwind-based method that adds less numerical viscosity and is more accurate on coarser grids compared to the PRICE method (32), but is computationally more expensive due to the characteristic decomposition that needs to be performed. The complete eigenstructure of the HME (12) was derived in Cai et al. (2013), so that the method of Osher and Solomon (31) can be implemented efficiently. In contrast, the PRICE method (32) does not rely on characteristic information and is thus computationally cheap, but its larger numerical viscosity requires finer grids to obtain comparable accuracy. This larger numerical viscosity can, however, be beneficial at boundary interfaces between different-order HME models, where it may suppress spurious oscillations caused by a mismatch in the number of variables; this is discussed further in Sect. 3.2.3.

Due to the simplicity of the BGK collision operator and the resulting collision term (14) in the HME model, the potentially stiff source term step (23) can be easily solved exactly and yields the analytical solution

33 $$\begin{aligned} w_i^{n+1} = w_i^{n+1,*}\exp \left( \Delta t S_M\right) , \end{aligned}$$

where $w_i^{n+1,*}$ is the solution of the transport step (24).

##### Remark 4

It is well known that the HME model (12) cannot be written in fully conservative form, since the evolution equation (11) for the highest-order moment is modified to ensure hyperbolicity. In the present formulation, primitive variables $\vec {w}_M=\left( \rho ,u,\theta ,f_3,\ldots ,f_M\right)$ are used, so only the density *ρ* satisfies a conservation law, which is a clear limitation. The advantage is that the system matrix $A_M(\vec {w}_M)$ (13) has a simple structure with analytical eigenvalues and eigenvectors (Cai et al. 2013). The model can also be written in conservative or partially conservative variables (Koellermeier and Torrilhon 2017), which is more physical but computationally more expensive for some schemes because the eigenvectors of the generalized Roe linearization are not explicitly given in that case. For strong shocks, conservative or partially conservative variables are preferable (Koellermeier and Torrilhon 2017); however, for the test cases in this paper, primitive variables give sufficiently accurate results, as also observed in Koellermeier and Torrilhon (2017) for similar test cases.

#### Spatial coupling at boundary interface

At the boundary interface between two subdomains that are modelled by an HME model of a different order, there is a mismatch in the number of variables and equations included in the model. In Verbiest and Koellermeier (2026), a padded buffer cell (PBC) coupling approach for the simulation of linear hierarchical moment equations was proposed. This approach was later applied to nonconservative shallow water moment models Verbiest and Koellermeier (2026), in which a path-conservative spatial coupling was proposed that is a particular case of the PBC coupling. In this paper, we propose a spatial coupling method that is based on the coupling proposed in Verbiest and Koellermeier (2026). The padded buffer cell is padded with additional zeros for path-conservation, as in Verbiest and Koellermeier (2026), but in contrast to Verbiest and Koellermeier (2026) only the fluctuations at the interface use the numerical viscosity matrix given by the PRICE method (32) and use the linear path (28), while (Verbiest and Koellermeier 2026) uses a Toumi-type path (Toumi 1992) at the boundary interfaces. The fluctuations away from the boundary interfaces are computed using the method of Osher and Solomon (31) that adds less numerical viscosity such that coarser grids can be used compared to the PRICE method (32). This is motivated by the observation that the artificial jump in the higher-order moments at the boundary interfaces creates small oscillations. Because of the form of the numerical viscosity matrix of the PRICE method (32), these oscillations will only affect the equations for the highest-order moments, whereas the characteristic decomposition that is performed in the numerical viscosity matrix of the method of Osher and Solomon (31) can cause the artificial jump to create oscillations in all variables.

**Fig. 1:**
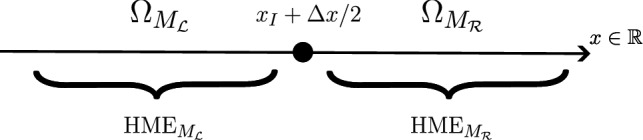
Domain decomposition example. Adapted from Verbiest and Koellermeier (2026)

Consider, as an example, a domain decomposition that consists of a single boundary interface located at $x=x_{I+1/2}{:}{=}x_I+\Delta x/2$, for some $I\in \{1,2,\ldots ,N_x-1\}$, that decomposes the domain into two subdomains $\Omega _\mathcal {L}$ and $\Omega _\mathcal {R}$ that are modelled by the $\text {HME}_{M_\mathcal {L}}$ and $\text {HME}_{M_\mathcal {R}}$, respectively, where $M_\mathcal {R}>M_\mathcal {L}\ge 2$. This is illustrated in Fig. 1.

Similarly to Verbiest and Koellermeier (2026), the fluctuations $D^\pm _{I+1/2}$ (25) at the boundary interface $x_{I+1/2}$ are computed using the padded state variable vector $\widetilde{w}_I^n\in \mathbb {R}^{M_\mathcal {R}+1}$ that pads the state variable vector $w_I^n\in \mathbb {R}^{M_\mathcal {L}+1}$ with additional zeros:

34 $$\begin{aligned} \widetilde{w}_I^n \, {:}{=} \, \left( (w_I^n)^T, 0, \ldots ,0 \right)^T. \end{aligned}$$

The path-conservative version of the PBC proposed in Verbiest and Koellermeier (2026) uses a Toumi-type path (Toumi 1992) at the boundary interface $x=x_{I+1/2}$ to compute the artificial jump caused by the padded zeros separately. In this paper, we use the linear path $\Phi _{\text {lin}}$ (28) because it allows a more efficient implementation and because only small differences were observed between the two paths in preliminary simulations. At the boundary interface $x=x_{I+1/2}$, the numerical viscosity matrix $Q_{\Phi _\mathrm{{lin}}}^\mathrm{{PRICE}}(\widetilde{w}_I^n,w_{I+1}^n)$ (32) is used to limit unphysical oscillations in the lower-order moments, so that the fluctuations $D^\pm _{I+\frac{1}{2}}$ (25) are given by

35 $$\begin{aligned}&D^\pm _{I+\frac{1}{2}} = \frac{1}{2}\left( A_{\Phi _\mathrm{{lin}}}(\widetilde{w}_I^n,w_{I+1}^n) (w_{I+1}^n-\widetilde{w}_I^n)\right. \nonumber \\& \quad \left. \pm Q_{\Phi _\mathrm{{lin}}}^\mathrm{{PRICE}}(\widetilde{w}_I^n,w_{I+1}^n) (w_{I+1}^n-\widetilde{w}_I^n) \right) , \end{aligned}$$

with generalized Roe linearization

36 $$\begin{aligned} A_{\Phi _\mathrm{{lin}}}(\widetilde{w}_I^n,w_{I+1}^n)=\int _{0}^1 A_{M_\mathcal {R}}(\Phi _\mathrm{{lin}}(s;\widetilde{w}_I^n,w_{I+1}^n))ds. \end{aligned}$$

The fluctuations at the remaining interfaces are computed using the numerical viscosity matrix $Q_{\Phi _\mathrm{{lin}}}^\mathrm{{OS}}(w_i^n,w_{i+1}^n)$ from the method of Osher and Solomon (31) that adds less numerical viscosity:

37 $$\begin{aligned}&D^\pm _{l+\frac{1}{2}} = \frac{1}{2}\left( A_{\Phi _\mathrm{{lin}}}(w_l^n,w_{l+1}^n) (w_{l+1}^n-w_l^n)\right. \nonumber \\& \quad \left. \pm Q_{\Phi _\mathrm{{lin}}}^\mathrm{{OS}}(w_l^n,w_{l+1}^n) (w_{l+1}^n-w_l^n) \right) , \quad \text {with} \end{aligned}$$

38 $$\begin{aligned}&A_{\Phi _\mathrm{{lin}}}(w_l^n,w_{l+1}^n)=\int _{0}^1 A_{M_\mathcal {L}}(\Phi _\mathrm{{lin}}(s;w_l^n,w_{l+1}^n))ds, \nonumber \\& \quad l = 1,2,\ldots ,I-1, \end{aligned}$$

in the left subdomain, and

39 $$\begin{aligned}&D^\pm _{r+\frac{1}{2}} = \frac{1}{2}\left( A_{\Phi _\mathrm{{lin}}}(w_r^n,w_{r+1}^n) (w_{r+1}^n-w_r^n) \right. \nonumber \\& \quad \left. \pm Q_{\Phi _\mathrm{{lin}}}^\mathrm{{OS}}(w_r^n,w_{r+1}^n) (w_{r+1}^n-w_r^n) \right) , \;\; \text {with} \end{aligned}$$

40 $$\begin{aligned}&A_{\Phi _\mathrm{{lin}}}(w_r^n,w_{r+1}^n)=\int _{0}^1 A_{M_\mathcal {R}}(\Phi _\mathrm{{lin}}(s;w_r^n,w_{r+1}^n))ds, \nonumber \\& \quad r = I + 1, \, I + 2,\ldots,N_x, \end{aligned}$$

in the right subdomain.

##### Remark 5

Setting the higher-order moments to zero in the padded state variable vector (34) at the boundary interface can be viewed as a particular boundary condition for the higher-order moments. We choose this zero padding in order to obtain a path-conservative scheme, at the expense of possible nonphysical reflections or oscillations at the boundary interface. Different coupling formulas lead to different boundary conditions for the higher-order moments at the boundary interfaces and may reduce these oscillations.

### Numerical approximation of domain decomposition criteria

To obtain a discretized set of domain decomposition criteria at discrete time $t_n$, where $n\in \{0,1,\ldots ,N_t\}$, numerical approximations of the gradients in the model-refinement model-error estimators (16), (17) and the model-coarsening model-error estimator (19) are computed at discrete times $t_n$ and $t_{n+1}$ and then inserted into the domain decomposition criteria (18) and (20). Evaluating (18) and (20) at both $t_n$ and $t_{n+1}$ results in a more accurate model-error estimation for the time step $t_n \rightarrow t_{n+1}$. It ensures that the used model order for simulating the time step $t_n \rightarrow t_{n+1}$ is sufficiently accurate at both end points of the time step interval $[t_n,t_{n+1}]$.

The evaluation of the model-error estimators (16), (17), and (19) at time $t_{n+1}$ requires first computing predictions $w_i^{n+1,\text {pred}}$ at time $t_{n+1}$, $i=1,2,\ldots ,N_x$, also for the approximation of the undefined higher-order moment in (17). In our implementation, they are efficiently computed by splitting Eq. (12) into a transport part and a collision part, simulating the transport step using a PVM (24) with numerical viscosity given by the cheaper PRICE method (32), compared to the more expensive method of Osher and Solomon (31), with only one quadrature node for the numerical approximation of the generalized Roe linearization (29), and then solving the collision part using the exact solution (33). Note that the predictions are computed using the PRICE method (32) on the same grid as the global finite volume updates that use the method of Osher and Solomon (31). Since the PRICE method (32) requires finer grids than the method of Osher and Solomon (31), the predictions are computationally cheap but less accurate. More accurate methods for the predictions are possible at the expense of a higher computational cost.

***Subdomain notation.*** The (still to be defined) discretized versions of the model-refinement criterion (18) and the model-coarsening criterion (20) decompose the domain $\Omega _x$ at discrete time $t_{n-1}$ into $K\le N_x$ non-overlapping subdomains

41 $$\begin{aligned} \Omega _x=\Omega _1^{n-1}\cup \Omega _2^{n-1}\cup \ldots \cup \Omega _K^{n-1}. \end{aligned}$$

For the remainder of this section, we will assume that the boundary interfaces at discrete time $t_{n-1}$ are given by the interfaces

42 $$\begin{aligned}&x_{(1)}^{n-1} = x_{I_1^{n-1}}+\Delta x/2,\quad x_{(2)}^{n-1}=x_{I_2^{n-1}}+\Delta x/2,\quad \ldots \quad ,\quad \nonumber \\& \quad x_{(K-1)}^{n-1}=x_{I_{K-1}^{n-1}}+\Delta x/2, \end{aligned}$$

where $I_1^{n-1},I_2^{n-1},\ldots ,I_{K-1}^{n-1}\in \{1,2,\ldots ,N_x-1\}$ and $x_{(1)}^{n-1}<x_{(2)}^{n-1}<\ldots <x_{(K-1)}^{n-1}$, so that

43 $$\begin{aligned} \Omega _l^{n-1}\cap \Omega _m^{n-1} = \delta _{l,m-1}x_{(l)}^{n-1}, \qquad \text {for } 1\le l < m \le K. \end{aligned}$$

Consider a subdomain $\Omega _k^{n-1}=\bigcup _{i = I_{k\,-1}^{n-1}}^{I_k^{n-1}-1} \mathcal {C}_i$, with $k\in \{1,\ldots ,K\}$, that is modelled by the $\text {HME}_{\overline{M}_k}$. For the boundary subdomains $\Omega _1^{n-1}$ and $\Omega _K^{n-1}$, we define $I_{0}^{n-1}=1$ and $I_{K}^{n-1}=N_x+1$. Consider a spatial cell $\mathcal {C}_i\subset \Omega _k^{n-1}$ and the discretized state variable vector $w_i^{n-1}$ at time $t_n$ in that cell, where $i\in \{I_{k\,-1}^{n-1},I_{k\,-1}^{n-1}+1,\ldots ,I_{k}^{n-1}-1\}$. The discretizations of the model-refinement criterion (18) and of the model-coarsening criterion (20) in cell $\mathcal {C}_i$ are discussed in Sects. 3.3.1 and 3.3.2, respectively.

#### Model-refinement criteria

Recall that the first model-refinement model-error estimator (16) is a special case of the second model-refinement model-error estimator (17) with $f_{\overline{M}_k+1} \equiv 0$. This motivates the following: the first model-refinement model-error estimator (16) is numerically evaluated at time $t_n$ using the discrete values $w_j^n$ and using the approximation $(f_{\overline{M}_k+1}^\text {approx})_j^n= 0$, where *j=i-1,i,i+1* to have fast numerical approximations. The second model-refinement model-error estimator (17) is numerically evaluated at time $t_{n+1}$ using the predicted values $w_j^{n+1,\text {pred}}$ and using the approximation $(f_{\overline{M}_k+1}^{\text {approx}})_j^{n+1}=(f_{\overline{M}_k+1})_j^{n+1,\text {pred}}$. We obtain the discrete approximations $\left( \mathcal {E}^{(1)}_{\overline{M}_k\rightarrow \overline{M}_k+2}\right) _i^n\in \mathbb {R}$ and $\left( \mathcal {E}^{(2)}_{\overline{M}_k\rightarrow \overline{M}_k+2}\right) _i^n\in \mathbb {R}$ to (16) and (17), respectively:

44 $$\begin{aligned} \left( \mathcal {E}^{(1)}_{\overline{M}_k\rightarrow \overline{M}_k+2}\right) _i^n \, {:}{=} \, \frac{\overline{M}_k+1}{2}\left( 2(f_{\overline{M}_k})_i^n\mathcal {D}_x u^n_i + (f_{\overline{M}_k-1})_i^n\mathcal {D}_x \theta ^n_i\right) , \end{aligned}$$

45 $$\begin{aligned} \begin{aligned}&\left( \mathcal {E}^{(2)}_{\overline{M}_k\rightarrow \overline{M}_k+2}\right) _i^n \, {:}{=} \, (\overline{M}_k+1)\mathcal {D}_x (f_{\overline{M}_k+1})_i^{n+1,\text {pred}}+\frac{\overline{M}_k+1}{2} \\& \quad \left( 2(f_{\overline{M}_k})_i^{n+1,\text {pred}}\mathcal {D}_x u^{n+1,\text {pred}}_i +(f_{\overline{M}_k-1})_i^{n+1,\text {pred}}\mathcal {D}_x\theta ^{n+1,\text {pred}}_i\right) , \end{aligned} \end{aligned}$$

where $\mathcal {D}_x g_i^n$ and $\mathcal {D}_x g_i^{n+1,\text {pred}}$ denote finite difference approximations to the *x-*derivative of the grid functions $g_j^n$ and $g_j^{n+1,\text {pred}}$, respectively, evaluated in cell $\mathcal {C}_i$. For the simulations in Sect. 4, we compute both the forward difference approximation $\mathcal {D}_xg_i^n = (g_{i+1}^n-g_i^n)/\Delta x$ and the backward difference approximation $\mathcal {D}_xg_i^n = (g_{i}^n-g_{i-1}^n)/\Delta x$ and compare their absolute values, and similarly for $g^{n+1,\text {pred}}_i$. The choice for one-sided difference approximations is motivated by the hyperbolicity of the HME model (12); characteristic waves with strengths proportional to the jumps of the state variables at the cell interfaces propagate into a cell from both sides. Rather than performing a full characteristic decomposition, which is computationally expensive, we approximate the spatial derivative by the corresponding forward and backward differences obtained from these interface jumps.

The fully discretized model-refinement criterion for each cell $\mathcal {C}_i$ in subdomain $\Omega _k^{n-1}$ is then summarized in the following definition.

##### Definition 3

*(Discretized model-refinement criterion)* Let subdomain $\Omega _k^{n-1}=\bigcup _{i = I_{k\,-1}^{n-1}}^{I_k^{n-1}} \mathcal {C}_i$, with $k\in \{1,\ldots ,K\}$, be modelled by the $\text {HME}_{\overline{M}_k}$ at time $t_{n-1}$ and set $I_{0}^{n-1}=1$ and $I_{K}^{n-1}=N_x+1$. The discretized model-refinement criterion $\left( \textrm{R}_{\overline{M}_k\rightarrow \overline{M}_k+2}\right) _i^n\in \{0,1\}$ in cells $\mathcal {C}_i$ in the subdomain $\Omega _k^{n-1}$ at time $t_n$ is defined as

46 $$\begin{aligned} \left( \textrm{R}_{\overline{M}_k\rightarrow \overline{M}_k+2}\right) _i^n \, {:}{=} \, \textbf{1}\!\left[ \max \left( \left| \left( \mathcal {E}^{(1)}_{\overline{M}_k\rightarrow \overline{M}_k+2}\right) _i^n\right| ,\left| \left( \mathcal {E}^{(2)}_{\overline{M}_k\rightarrow \overline{M}_k+2}\right) _i^n\right| \right)> \epsilon _\textrm{R}\right] , \end{aligned}$$

for $i=I_{k-\,1}^{n-1},I_{k-\,1}^{n-1}+1,\ldots ,I_{k}^{n-1}-1$, where $\left( \mathcal {E}^{(1)}_{\overline{M}_k\rightarrow \overline{M}_k+2}\right) _i^n$ and $\left( \mathcal {E}^{(2)}_{\overline{M}_k\rightarrow \overline{M}_k+2}\right) _i^n$ are defined by (44) and (45), respectively, and where $\epsilon _\textrm{R}>0$ is a chosen refinement threshold. Here, $\textbf{1}\!\left[ \cdot \right]$ is the indicator function that is defined as $\textbf{1}\!\left[ A\right] =1$ if the statement *A* holds and $\textbf{1}\!\left[ A\right] =0$ otherwise. $\left( \textrm{R}_{\overline{M}_k\rightarrow \overline{M}_k+2}\right) _i^n=1$ signals model refinement in cell $\mathcal {C}_i$.

#### Model-coarsening criteria

Analogous to the discretization of the model-refinement criterion, the model-coarsening model-error estimator (19) is numerically evaluated at discrete times $t_n$ and $t_{n+1}$ by replacing the spatial derivatives by finite difference approximations, yielding the two discrete approximations $\left( \mathcal {E}^{(1)}_{\overline{M}_k\rightarrow \overline{M}_k-2}\right) _i^n\in \mathbb {R}$ and $\left( \mathcal {E}^{(2)}_{\overline{M}_k\rightarrow \overline{M}_k-2}\right) _i^n\in \mathbb {R}$ (assuming $\overline{M}_k\ge 4$)

47 $$\begin{aligned} \begin{aligned}&\left( \mathcal {E}^{(1)}_{\overline{M}_k\rightarrow \overline{M}_k-2}\right) _i^n \, {:}{=} \, (\overline{M}_k-1)\mathcal {D}_x (f_{\overline{M}_k-1})_i^n + \frac{\overline{M}_k-1}{2} \\& \quad \left( 2(f_{\overline{M}_k-2})^n_i\mathcal {D}_x u^n_i+(f_{\overline{M}_k-3})_i^n\mathcal {D}_x\theta ^n_i\right) ,\end{aligned} \end{aligned}$$

48 $$\begin{aligned} \begin{aligned}&\left( \mathcal {E}^{(2)}_{\overline{M}_k\rightarrow \overline{M}_k-2}\right) _i^n \, {:}{=} \, (\overline{M}_k-1)\mathcal {D}_x (f_{\overline{M}_k-1})^{n+1,\text {pred}}_i \\&\quad + \frac{\overline{M}_k-1}{2}\left( 2(f_{\overline{M}_k-2})_i^{n+1,\text {pred}}\mathcal {D}_x u_i^{n+1,\text {pred}} \right. \\& \quad \left. +(f_{\overline{M}_k-3})_i^{n+1,\text {pred}}\mathcal {D}_x\theta ^{n+1,\text {pred}}_i\right) . \end{aligned} \end{aligned}$$

The fully discrete model-coarsening criterion is summarized in the following definition.

##### Definition 4

*(Discretized model-coarsening criterion)* Let subdomain $\Omega _k^{n-1}=\bigcup _{i = I_{k-1}^{n-1}}^{I_k^{n-1}-1} \mathcal {C}_i$, with $k\in \{1,\ldots ,K\}$, be modelled by the $\text {HME}_{\overline{M}_k}$ at time $t_{n-1}$ and set $I_{0}^{n-1}=1$ and $I_{K}^{n-1}=N_x+1$. If $\overline{M}_k\ge 4$, the discretized model-coarsening criterion $\left( \textrm{C}_{\overline{M}_k\rightarrow \overline{M}_k-2}\right) _i^n\in \{0,1\}$ in cells $\mathcal {C}_i$ in the subdomain $\Omega _k^n$ at time $t_n$ is defined as

49 $$\begin{aligned} \left( \textrm{C}_{\overline{M}_k\rightarrow \overline{M}_k-2}\right) _i^n {:}{=} \textbf{1}\!\left[ \max \left( \left| \left( \mathcal {E}^{(1)}_{\overline{M}_k\rightarrow \overline{M}_k-2}\right) _i^n\right| ,\left| \left( \mathcal {E}^{(2)}_{\overline{M}_k\rightarrow \overline{M}_k-2}\right) _i^n\right| \right) < \epsilon _\textrm{C}\right] , \end{aligned}$$

for $i=I_{k-\,1}^{n-1},I_{k-\,1}^{n-1}+1,\ldots ,I_{k}^{n-1}-1$, where $\left( \mathcal {E}^{(1)}_{\overline{M}_k\rightarrow \overline{M}_k-2}\right) _i^n$ and $\left( \mathcal {E}^{(2)}_{\overline{M}_k\rightarrow \overline{M}_k-2}\right) _i^n$ are defined by (47) and (48), respectively, and where $\epsilon _\textrm{C}>0$ is a chosen coarsening threshold. Here, $\textbf{1}\!\left[ \cdot \right]$ is the indicator function that is defined as $\textbf{1}\!\left[ A\right] =1$ if the statement *A* holds and $\textbf{1}\!\left[ A\right] =0$ otherwise. $\left( \textrm{C}_{\overline{M}_k\rightarrow \overline{M}_k-2}\right) _i^n=1$ signals model coarsening in cell $\mathcal {C}_i$.

Note that special care needs to be taken to compute the finite difference approximations to the gradients at the boundary interfaces. The finite difference approximations at the boundary interfaces of moments that are not defined in one of the two bordering subdomains are set to zero to eliminate the artificial jump in the higher-order moments from the approximations. To be more precise, assume that $k\in \{2,3.\ldots ,K-1\}$, such that $\Omega _k^{n-1}$ has two neighbouring subdomains $\Omega _{k-1}^{n-1}$ and $\Omega _{k+1}^{n-1}$ that are modelled by the $\text {HME}_{\overline{M}_{k-1}}$ and the $\text {HME}_{\overline{M}_{k+1}}$, respectively. If $\overline{M}_{k-1}< \overline{M}_k$, then the moments $f_{\overline{M}_k-1}$ and $f_{\overline{M}_k}$ are not defined in cell $\mathcal {C}_{I_{k\,-1}-1}$. The gradients in the moments $f_{\overline{M}_k-1}$ and $f_{\overline{M}_k}$ appearing in (44) and (45) are set to zero when the backward finite difference approximation is used for discretizing the gradients. If $\overline{M}_{k+1}< \overline{M}_k$, then the moments $f_{\overline{M}_k-1}$ and $f_{\overline{M}_k}$ are not defined in cell $\mathcal {C}_{I_k}$. The gradients in the moments $f_{\overline{M}_k-1}$ and $f_{\overline{M}_k}$ appearing in (44) and (45) are set to zero when the forward finite difference approximation is used for discretizing the gradients. This procedure is also applied to the corresponding gradients appearing in (47) and (48).

### Algorithm for model-adaptive simulation of HME

Finally, the complete algorithm for the model-adaptive simulation of the HME (12) is summarized in Algorithm 1. The algorithm combines the discretized domain decomposition criteria proposed in definitions 3 and 4, with the spatial coupling proposed in Sect. 3.2. For a practical implementation, a maximum order $M_{\text {max}}\ge 2$ is chosen.

**Algorithm 1:**
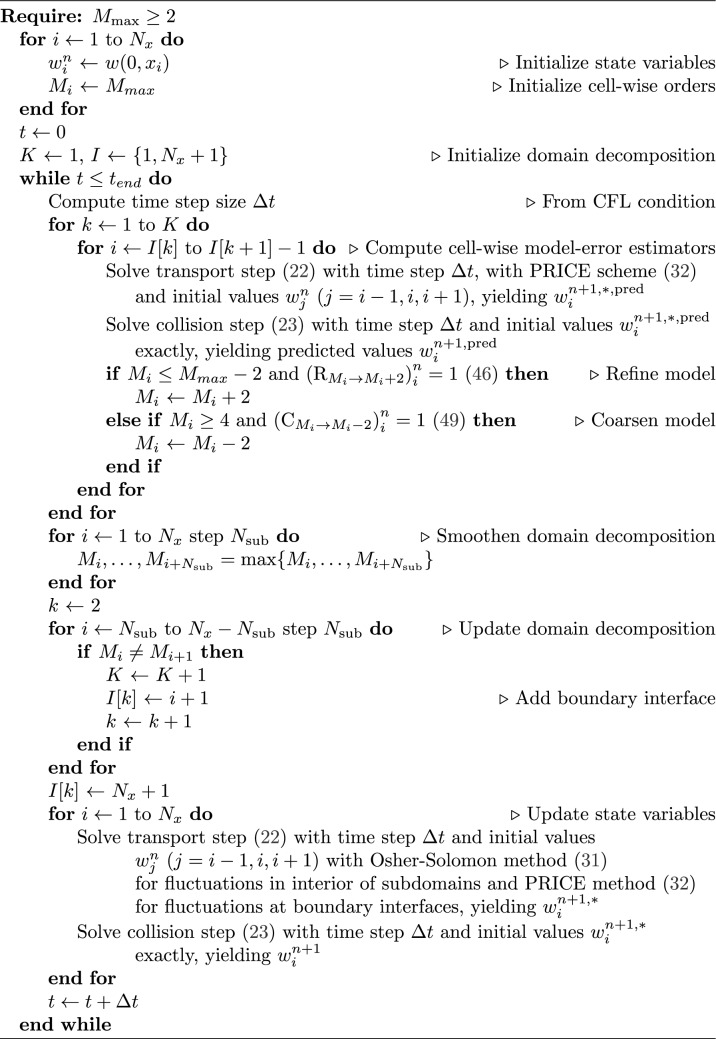
**(A-HME)** Algorithm for the model-adaptive simulation of HME

**Fig. 2:**
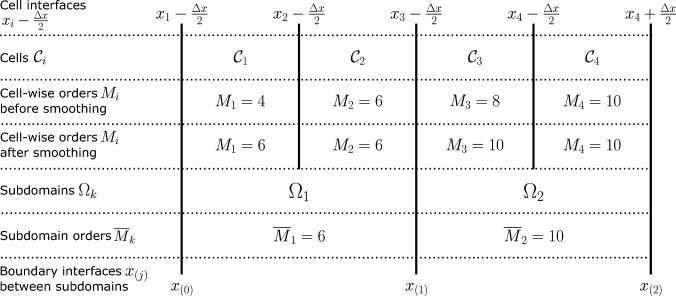
Smoothing of the domain decomposition with minimum subdomain size $N_{\text {sub}}=2$ on a domain with $N_x=4$ cells. The superscripts denoting time are omitted

The discretized model-refinement criterion (46) and the discretized model-coarsening criterion (49) result in a domain decomposition that assigns to each cell $\mathcal {C}_i$, $i=1,\ldots ,N_x$ an updated cell-wise model-order $M_i$. At each boundary interface, an artificial jump in the highest-order moments is created, which causes small oscillations at the boundary interfaces. To avoid that the oscillations created at the boundary interfaces interact with each other, we propose a smoothed domain decomposition in which each subdomain consists of at least $N_{\text {sub}}>1$ cells, where $N_{\text {sub}}$ is called the minimum subdomain size parameter. For an efficient implementation, we fix the possible boundary interfaces and compute the maximum cell-wise model-order $M_i$ in-between every pair of subsequent boundary interfaces. This is illustrated in Fig. 2 for a discretized domain with $N_x=4$ cells and with minimum subdomain size $N_{\text {sub}}=2$. Note that for the remainder of this paper, we will denote a model-adaptive simulation of the HME model by the short notation A-HME.

## Numerical simulations

In this section, the new model-adaptive method A-HME summarized in Algorithm 1 is numerically tested for different values of the model-coarsening threshold parameter $\epsilon _\textrm{C}$ and the model-refinement threshold parameter $\epsilon _\textrm{R}$, different values for the minimum subdomain size parameter $N_\text {sub}$, and for two different Knudsen numbers $\text {Kn}=0.05$ and $\text {Kn}=0.5$. The purpose of this section is to analyze the accuracy and stability of the model-adaptive method A-HME and the effect of the physical parameter Kn and the method parameters $\epsilon _\textrm{C},\epsilon _\textrm{R},N_\text {sub}$ on the accuracy and the stability.

For all simulation results using the A-HME, the maximum order was set to $M_{\text {max}}=12$. For the HME and the A-HME simulations, the grid size was set to *Δ x = 0.003* and the time step *Δ t* was adaptively chosen with CFL number 0.7. The relaxation time *τ* was chosen as $\tau =\frac{\text {Kn}}{\rho }$.

### Remark 6

We present A-HME results for a representative subset of minimum subdomain sizes $N_\text {sub}\in \{5,10,20\}$, threshold value combinations

50 $$\begin{aligned} & (\epsilon _\textrm{C},\epsilon _\textrm{R})\in \\& \,\, \{({1\times 10^{-4}},{1.5 \times 10^{-4}}),({3 \times 10^{-4}},{4.5 \times 10^{-4}}),({1 \times 10^{-3}},{1.5 \times 10^{-3}})\} \end{aligned}$$

and test cases to keep the presentation concise. Additional simulations show qualitatively similar behavior. The threshold parameters were chosen based on preliminary numerical experiments.

### Density shock wave colliding with smooth density wave

**Fig. 3:**
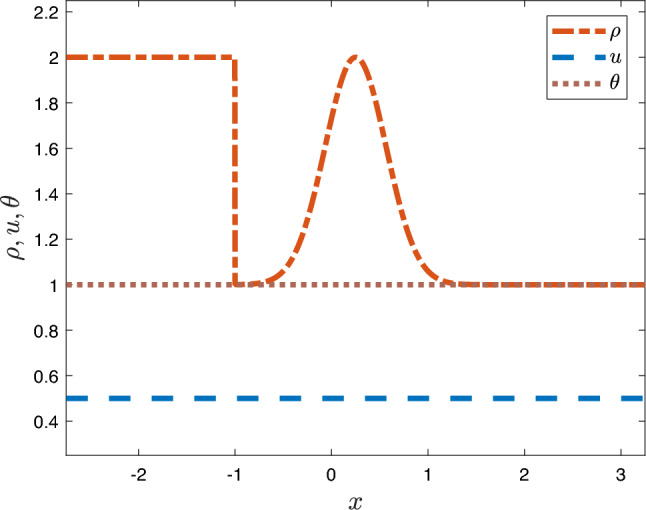
Initial conditions of density shock wave colliding with smooth density wave. Initial density *ρ (0,x)* (red, dash-dotted), initial velocity *u(0,x)* (blue, dashed), and initial temperature *θ (0,x)* (brown, dotted)

**Fig. 4:**
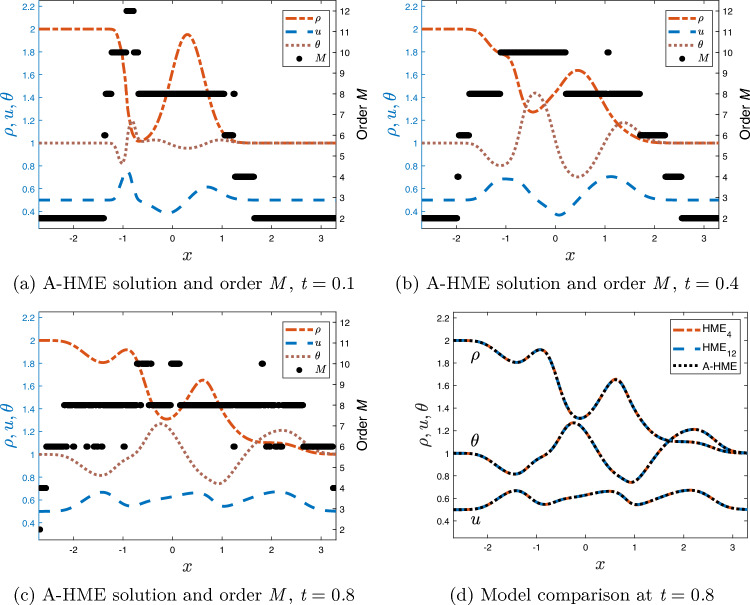
Density shock wave colliding with smooth density wave, with Kn = 0.05 and $t\in \{0.1,0.4,0.8\}$. For the A-HME, the minimum subdomain size parameter is set to $N_{\text {sub}}=10$, and the threshold values are set to $\epsilon _\textrm{C}={3.0 \times 10^{-4}}$ and $\epsilon _\textrm{R}={4.5 \times 10^{-4}}$. (**a**)-(**c**): Density *ρ* (red, dash-dotted), velocity *u* (blue, dashed), temperature *θ* (brown, dotted), and order *M* in each cell (black dots). All variables *ρ ,u* and *θ* were computed using the A-HME. (**d**): Comparison of the lower-order $\text {HME}_{4}$ (red, dash-dotted), the highest-order $\text {HME}_{12}$ (blue, dashed) and the A-HME (black, dotted). The domain decomposition assigns higher orders to regions with larger gradients of *ρ ,u* and *θ*, and the maximum order $M_{\text {max}}=12$ is only assigned to the front of the shock wave at time *t=0.1*. Furthermore, the A-HME, $\text {HME}_{4}$ and the $\text {HME}_{12}$ yield similar numerical results

**Fig. 5:**
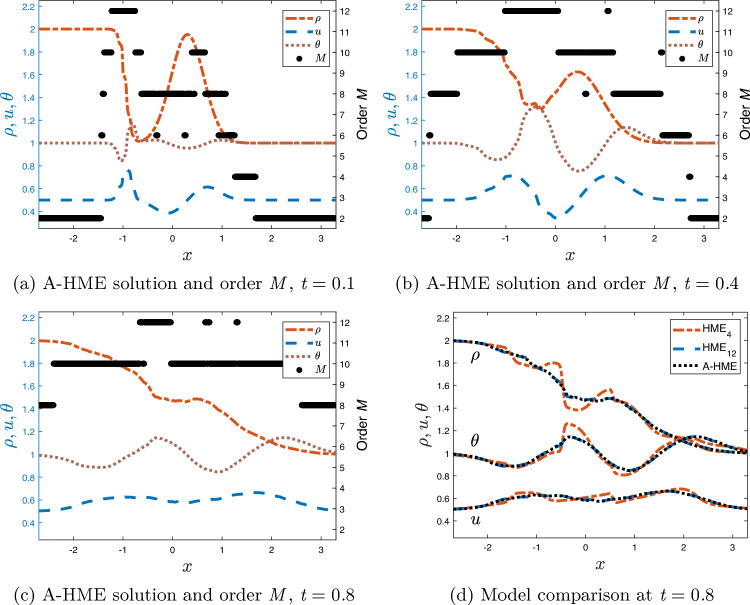
Density shock wave colliding with smooth density wave, with Kn = 0.5 and $t\in \{0.1,0.4,0.8\}$. For the A-HME, the minimum subdomain size parameter is set to $N_{\text {sub}}=10$, and the threshold values are set to $\epsilon _\textrm{C}={3.0 \times 10^{-4}}$ and $\epsilon _\textrm{R}={4.5 \times 10^{-4}}$. (**a**)-(**c**): Density *ρ* (red, dash-dotted), velocity *u* (blue, dashed), temperature *θ* (brown, dotted), and order *M* in each cell (black dots). All variables *ρ ,u* and *θ* were computed using the A-HME. (**d**): Comparison of the lower-order $\text {HME}_{4}$ (red, dash-dotted), the highest-order $\text {HME}_{12}$ (blue, dashed) and the A-HME (black, dotted). The domain decomposition assigns higher orders to regions with larger gradients of *ρ ,u* and *θ*. Moreover, higher orders are assigned compared to $\text {Kn}=0.05$ (Fig. 4). Furthermore, the A-HME yields numerical results that are very close to the highest-order $\text {HME}_{12}$ compared to the lower-order $\text {HME}_{4}$

To analyze the performance of the A-HME, we consider as a first toy test case a density shock wave colliding with a smooth density wave, similar to Verbiest and Koellermeier (2026). The initial conditions are

51 $$\begin{aligned} \begin{aligned} \rho (0,x)&= {\left\{ \begin{array}{ll} 2, & x \le -1, \\ 1 + \exp \left( -5(x-0.25)^2\right) , & x> 1, \end{array}\right. }\\ u(0,x)&=0.5, \quad T(0,x)=1, \quad f_i(0,x)=0\;\;\forall i\ge 3, \end{aligned} \end{aligned}$$

for an infinitely long domain $\Omega _x = \mathbb {R}$, where the results are plotted in *[-2.7,3.3]*. The initial density profile, shown in Fig. 3, consists of both a discontinuity and a smooth part to analyze how the A-HME, and in particular the domain decomposition, handles discontinuities and smooth waves. The initial velocity was chosen to be non-zero to remove the symmetry of the smooth wave and add complexity to the flow. The results for the A-HME have been obtained by using the minimum subdomain size $N_{\text {sub}}=10$ and the threshold values $\epsilon _\textrm{C}={3.0 \times 10^{-4}}$ and $\epsilon _\textrm{R}={4.5 \times 10^{-4}}$. In Figs. 4 and 5, the results of the model-adaptive method A-HME are compared with the highest-order $\text {HME}_{12}$ and the lower-order $\text {HME}_4$, which was chosen to illustrate the model differences. A complete comparison of the errors and the runtimes of all (even order) HME models up to maximum order $M_\text {max}=12$ is given in Sect. 4.1.3.

The simulations were run with end time $t_{\text {end}}=0.8$ and snapshots of the numerical solutions are shown at times $t\in \{0.1,0.4,0.8\}$. The first aim of this test case is to analyze the accuracy of the model-refinement criterion (46) and the model-coarsening criterion (49) in capturing the regions of the rarefied gas domain that require a higher-order HME model and regions that can be modelled sufficiently accurately by a lower-order HME model. Moreover, we are interested in the evolution of the domain decomposition in time. The second aim of this test case is to quantify the computational speedup the A-HME obtains, compared to a simulation with an $\text {HME}$ model in the entire domain that reaches a similar accuracy as the A-HME.

#### Density shock wave colliding with smooth density wave with Kn = 0.05

The results for small $\text {Kn} = 0.05$ are shown in Fig. 4. In Fig. 4a-c, the order *M* in each cell is shown at different times, together with the density *ρ*, velocity *u* and temperature *θ*. At time *t=0.1* (Fig. 4a), the domain decomposition assigns the maximum order $M_{\text {max}}=12$ to the front of the shock wave at *x≈ -0.8* and a lower order *M=8* to the smooth wave. At time *t=0.4* (Fig. 4b), the smooth wave and the shock wave have started to collide and the subdomain *x∈ [-1,0]* in which this occurs is assigned the order *M=10*, the highest order appearing in the entire domain at that time. At time *t=0.8* (Fig. 4c), the shock wave and the smooth wave have collided and the shock wave has been smoothed out slightly, resulting in a domain decomposition that has become more uniform. It is observed in Fig. 4a-c that large local gradients of the state variables *ρ ,u* and *θ* lead to higher orders in the domain decomposition, as expected. Finally, a model-comparison of the lower-order $\text {HME}_4$, the highest-order $\text {HME}_{12}$ and the A-HME at *t=0.8* is shown in Fig. 4d. For this small Kn, the flow is near equilibrium, and the difference between the different models is small for all variables and cannot be visually distinguished.

#### Density shock wave colliding with smooth density wave with Kn = 0.5

Now, we consider the test case (51) with large Kn = 0.5. The results of this test case are shown in Fig. 5. The results are analyzed and compared with the results of the test case with small $\text {Kn}=0.05$ shown in Fig. 4. Figure 5a-c display the orders *M* in each cell at different times. At time *t=0.1* (Fig. 5a), the domain decomposition assigns the maximum order $M_\text {max}=12$ to the entire subdomain *x∈ [-1.3,-0.7]* affected by the shock wave and assigns orders *M=8* and *M=10* to the smooth wave. At time *t=0.4* (Fig. 5b), the smooth wave and the shock wave have started to collide and the subdomain *x∈ [-1.1,0.1]* in which this occurs is assigned the maximum order $M_\text {max}=12$. At time *t=0.8* (Fig. 5c), the shock wave and the smooth wave have collided and the shock wave has been smoothed out considerably, resulting in a domain decomposition that has become more uniform. Although the local gradients of *ρ ,u* and *θ* have decreased in magnitude, the domain decomposition still assigns the maximum order $M_\text {max}=12$ to some subdomains and the order *M=10* to most of the remaining subdomains.

Compared to the results of the test case with small $\text {Kn} = 0.05$ in Sect. 4.1.1, the test case with $\text {Kn} = 0.5$ yields domain decompositions with generally higher orders, which is expected since there is more non-equilibrium for $\text {Kn}=0.5$ due to the larger Kn.

Finally, a model-comparison of the lower-order $\text {HME}_4$, the highest-order $\text {HME}_{12}$ and the A-HME at *t=0.8* is shown in Fig. 5d. While the difference between the lower-order $\text {HME}_4$ and the highest-order $\text {HME}_{12}$ is considerable for all variables, the difference between the highest-order $\text {HME}_{12}$ and the A-HME is almost non-existent, indicating the desired accuracy of the A-HME.

#### Error and runtime comparison

The aim of this paper is to accelerate the numerical simulations of the HME by only using higher-order HME models in subdomains where it is necessary to reach sufficient accuracy and by using lower-order HME models in subdomains where they achieve sufficient accuracy. The estimated relative model errors and the relative runtimes (both with respect to the highest-order $\text {HME}_{12}$) for both small $\text {Kn}=0.05$ and large $\text {Kn}=0.5$ are given in Table 1. Note that the implementation of the A-HME is not optimized and that larger speedups can potentially be obtained. The model errors are computed by summing the relative 2-norm errors of the density *ρ*, velocity *u* and temperature *θ* with respect to the highest-order $\text {HME}_{12}$. Note that the 2-norm of the error alone does not fully characterize the approximation quality. In particular, the A-HME might resolve local non-equilibrium features that are not captured by a low-order HME model, even when they produce comparable 2-norm errors.

**Table 1:** Relative errors and relative runtimes (both with respect to the highest-order $\text {HME}_{12}$) of simulation of density shock wave colliding with smooth density wave for $\text {HME}_M$, *M=2,4,6,8,10*, and for the adaptive A-HME with $N_\text {sub}=10$, $\epsilon _\textrm{C}={3.0 \times 10^{-4}}$, and$\epsilon _\textrm{R}={4.5 \times 10^{-4}}$, at two values of Kn (0.05, 0.5). The A-HME reduces the computational time compared to a non-adaptive HME model simulation that reaches similar accuracy, especially for the larger Knudsen number $\text {Kn}=0.5$.

Model	Kn = 0.05		Kn = 0.5	
	Relative error	Relative runtime	Relative error	Relative runtime
HME$_2$	1.5$\times 10^{-1}$	0.07	3.2$\times 10^{-1}$	0.07
HME$_4$	3.9$\times 10^{-3}$	0.14	1.1$\times 10^{-1}$	0.14
HME$_6$	9.5$\times 10^{-4}$	0.26	5.5$\times 10^{-2}$	0.26
HME$_8$	**3.5**$\times {\textbf {10}}^{\mathbf{-4}}$	**0.43**	3.0$\times 10^{-2}$	0.42
HME$_{10}$	2.4$\times 10^{-5}$	0.68	**1.3**$\times {\textbf {10}}^{\mathbf{-2}}$	**0.66**
A-HME	**3.6**$\times {\textbf {10}}^{\mathbf{-4}}$	**0.40**	**1.3**$\times {\textbf {10}}^{\mathbf{-2}}$	**0.56**

For the test case with small $\text {Kn}=0.05$, the A-HME reaches nearly the same accuracy as the $\text {HME}_8$, with a small relative error of the order $O\left( {10^{-4}}\right)$, while slightly reducing the computational time. The more accurate $\text {HME}_{10}$ has an error of the order $O\left( 10^{-5}\right)$, but the A-HME reduces the computational time by approximately 40 percent with respect to the $\text {HME}_{10}$.

The results for the test case with large $\text {Kn}=0.5$ are more pronounced. The A-HME yields the smallest error together with the $\text {HME}_{10}$, but reduces the computational time by approximately 15 percent with respect to the $\text {HME}_{10}$.

It is important to note that both the accuracy and the runtime of the HME depend on the minimum subdomain size parameter $N_{\text {sub}}$ and the threshold parameters $\epsilon _\textrm{C}$ and $\epsilon _\textrm{R}$. The values for these parameters were chosen based on observations. For optimal results, the effect of the parameters on the simulation results of the A-HME should be studied in more detail. In the next section, a shock tube test case is considered and the simulation results using the A-HME with varying $N_{\text {sub}}$, $\epsilon _\textrm{C}$ and $\epsilon _\textrm{R}$ are compared.

### Shock tube

As second test case, we consider a shock tube with initial conditions

52 $$\begin{aligned} \rho (0,x) = {\left\{ \begin{array}{ll} 7, & x \le 0 \\ 1, & x> 0 \end{array}\right. }, \quad u(0,x)=0, \quad T(0,x)=1, \quad f_i(0,x)=0\;\;\forall i\ge 3, \end{aligned}$$

in the spatial domain $x\in \Omega _x = [-1.5,1.5]$. The results are compared with the results of a discrete velocity method (DVM) (Mieussens 2000), provided as reference solutions. The DVM has been validated for a shock tube with experimental data in Mieussens (2000). The A-HME is run with three different minimum subdomain sizes $N_{\text {sub}}\in \{5,10,20\}$ and three different threshold parameter value combinations

53 $$\begin{aligned} \begin{aligned} (\epsilon _\textrm{C},\epsilon _\textrm{R})_\textrm{s}&=({1 \times 10^{-4}},{1.5 \times 10^{-4}}),\\ (\epsilon _\textrm{C},\epsilon _\textrm{R})_\textrm{m}&=({3 \times 10^{-4}},{4.5 \times 10^{-4}}), \\(\epsilon _\textrm{C},\epsilon _\textrm{R})_\textrm{l}&=({1 \times 10^{-3}},{1.5 \times 10^{-3}}), \end{aligned} \end{aligned}$$

that represent small threshold values $(\epsilon _\textrm{C},\epsilon _\textrm{R})_\textrm{s}$, moderate threshold values $(\epsilon _\textrm{C},\epsilon _\textrm{R})_\textrm{m}$, and large threshold values $(\epsilon _\textrm{C},\epsilon _\textrm{R})_\textrm{l}$, respectively.

The end time of the simulation is $t_{\text {end}}=0.3$. The A-HME is run with the different parameters and for both $\text {Kn}=0.05$ and $\text {Kn}=0.5$. For comparison, the simulation results of the highest-order $\text {HME}_{12}$ and the lower-order $\text {HME}_4$ are added. As in the previous test case in Sect. 4.1, the lower-order HME model was chosen to illustrate the model differences. A complete comparison of the errors and the runtimes of all (even order) HME models up to maximum order $M_\text {max}=12$ is given in Tables 2 and 3, which will be discussed later in this section.

#### Shock tube with Kn = 0.05

**Fig. 6:**
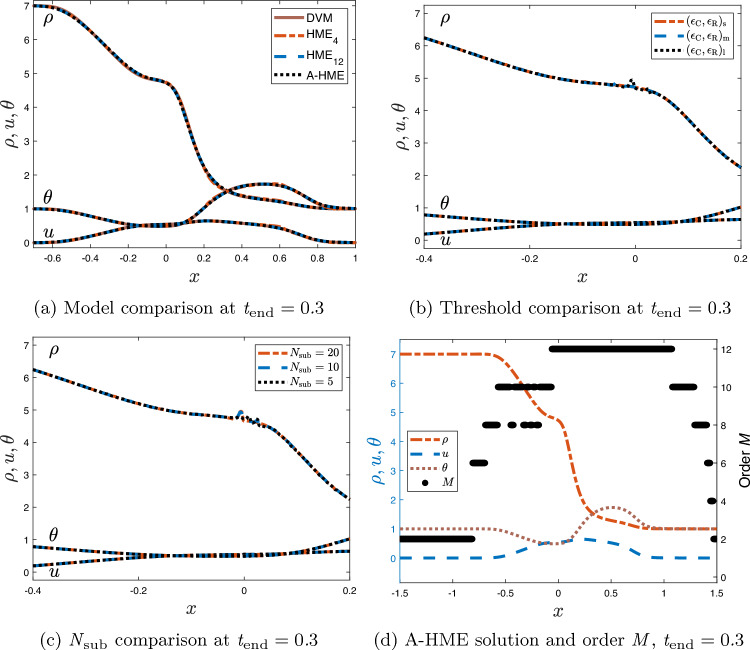
Shock tube with Kn = 0.05 and $t_{\text {end}}=0.3$. All plots show solutions for the density *ρ*, velocity *u* and temperature *θ*. (**a**): Model comparison of $\text {HME}_{4}$ (red, dash-dotted), $\text {HME}_{12}$ (blue, dashed), A-HME with minimum subdomain size $N_{\text {sub}}=10$ and with small threshold values $(\epsilon _\textrm{C},\epsilon _\textrm{R})_\textrm{s}=({1 \times 10^{-4}},{1.5 \times 10^{-4}})$ (black, dotted), and DVM reference solutions (brown, solid). (**b**): Comparison of the A-HME solutions that use minimum subdomain size $N_{\text {sub}}=10$ for different threshold values $(\epsilon _\textrm{C},\epsilon _\textrm{R})_\textrm{s}=({1 \times 10^{-4}},{1.5 \times 10^{-4}})$ (small thresholds, red dash-dotted line), $(\epsilon _\textrm{C},\epsilon _\textrm{R})_\textrm{m}=({3 \times 10^{-4}},{4.5 \times 10^{-4}})$ (moderate thresholds, blue dashed line) and $(\epsilon _\textrm{C},\epsilon _\textrm{R})_\textrm{l}=({1 \times 10^{-3}},{1.5 \times 10^{-3}})$ (large thresholds, black dotted line). (**c**): Comparison of A-HME solutions using the large threshold values $(\epsilon _\textrm{C},\epsilon _\textrm{R})_\textrm{l}$ for minimum subdomain sizes $N_\text {sub}=20$ (red, dash-dotted), $N_\text {sub}=10$ (blue, dashed), and $N_\text {sub}=5$ (black, dotted). (**d**): Final domain decomposition of the A-HME simulation with $N_\text {sub}=10$ and with threshold values $(\epsilon _\textrm{C},\epsilon _\textrm{R})_\textrm{s}$, together with the A-HME solutions of *ρ* (red, dash-dotted), *u* (blue, dashed), and *θ* (black, dotted). An oscillation is visible around *x≈ 0* for *ρ* in the A-HME solutions with moderate threshold values $(\epsilon _\textrm{C},\epsilon _\textrm{R})_\textrm{m}$ and large threshold values $(\epsilon _\textrm{C},\epsilon _\textrm{R})_\textrm{l}$, regardless of the minimum subdomain size $N_\text {sub}$. The oscillation is largest for the large threshold values $(\epsilon _\textrm{C},\epsilon _\textrm{R})_\textrm{l}$

The results for small $\text {Kn}=0.05$ are shown in Fig. 6. Figure 6a shows a comparison of the lower-order $\text {HME}_4$, the highest-order $\text {HME}_{12}$, the adaptive A-HME with minimum subdomain size $N_\text {sub}=10$ and small threshold values $(\epsilon _\textrm{C},\epsilon _\textrm{R})_\textrm{s}$, and the DVM. For this small Kn, the flow is near equilibrium, and all models yield similar results. In particular, the A-HME yields accurate results compared to the reference DVM results, comparable to the accuracy of the highest-order $\text {HME}_{12}$ and slightly more accurate than the lower-order $\text {HME}_4$.

Figure 6b compares the results of the A-HME with minimum subdomain size $N_\text {sub}=10$ for the different threshold values. Only for the large threshold values $(\epsilon _\textrm{C},\epsilon _\textrm{R})_\textrm{l}$, an oscillation is visible in the density *ρ* around *x≈ 0*. This shows that smaller thresholds are effective at decreasing potential oscillations at model interfaces in this case.

To investigate the effect of the minimum subdomain size parameter on this oscillation, Fig. 6c compares the A-HME results with large threshold values $(\epsilon _\textrm{C},\epsilon _\textrm{R})_\textrm{l}$ for different minimum subdomain sizes $N_\text {sub}\in \{5,10,20\}$. The oscillation remains present when the minimum subdomain size is increased to $N_\text {sub}=20$, so that we conclude that smoothing the domain decomposition over more cells does not yield an improvement in this case.

Finally, the domain decomposition of the A-HME with $N_\text {sub}=10$ and small threshold values $(\epsilon _\textrm{C},\epsilon _\textrm{R})_\textrm{s}$ at $t_{\text {end}}=0.3$ is shown in Fig. 6d, which shows the order *M* in each cell together with *ρ*, *u* and *θ*. In general, the domain decomposition assigns higher orders to regions with larger gradients of *ρ*, *u* and *θ*.

**Fig. 7:**
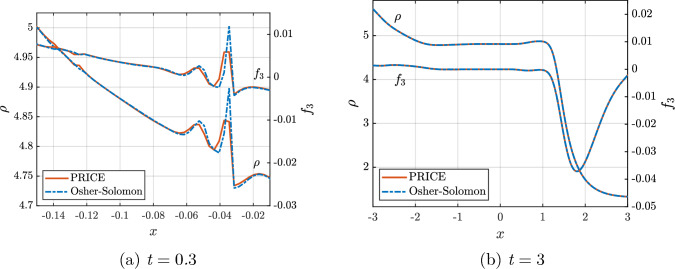
Interface method comparison for A-HME with minimum subdomain size $N_{\text {sub}}=10$ and moderate threshold values $(\epsilon _\textrm{C},\epsilon _\textrm{R})_\textrm{m}=({3 \times 10^{-4}},{4.5 \times 10^{-4}})$ for shock tube with Kn = 0.05, at (**a**) *t=0.3* and (**b**) *t=3*. The PRICE method (red, solid) or the method of Osher and Solomon (blue, dash-dotted) are used at the boundary interfaces, and they are compared for the density *ρ* and the moment $f_3$. At time *t=0.3*, the PRICE method results in a smaller oscillation compared to the method of Osher and Solomon. The oscillations are damped and vanish for longer simulation times

For different discretizations at the interfaces, the stability of the A-HME with $N_{\text {sub}}=10$ and moderate threshold values $(\epsilon _\textrm{C},\epsilon _\textrm{R})_\textrm{m}$ for the shock tube with $\text {Kn}=0.05$ is analysed in Fig. 7. Figure 7a shows oscillations in the density *ρ* and the moment $f_3$ at the early time *t=0.3*. A similar oscillation was observed in the other variables as well, but these plots were omitted for brevity. It is observed that the PRICE method (32) results in an oscillation with a smaller magnitude compared to the method of Osher and Solomon (31). However, the PRICE method (32) at the boundary interfaces resulted in small artifacts, seen around *x≈ -0.125*, for example. Figure 7b shows the results for an extended simulation time *t=3* to study the long-term stability of the A-HME. The oscillations are no longer present for this extended simulation time. Moreover, both interface coupling methods yield similar results that cannot be visibly distinguished.

Relative errors of the different models with respect to the DVM simulations were computed by summing the relative errors in the density *ρ*, velocity *u*, and temperature *θ*. The relative errors for the different HME models and for A-HME models with different values of the minimum subdomain size parameter $N_{\text {sub}}$ and the threshold parameters $\epsilon _\textrm{C}$ and $\epsilon _\textrm{R}$ are given in Table 2, for small $\text {Kn} = 0.05$. As observed in Fig. 6a, the smallest threshold values $(\epsilon _{\textrm{C}},\epsilon _{\textrm{R}})_\textrm{s}$ yield accurate results, close to the error of the highest-order $\text {HME}_{12}$. Moreover, increasingly accurate results are obtained with increasing minimum subdomain sizes $N_{\text {sub}}$, as expected because increasing the minimum subdomain size $N_{\text {sub}}$ increases the order *M* in some cells. For the moderate threshold values $(\epsilon _{\textrm{C}},\epsilon _{\textrm{R}})_\textrm{m}$, the error approaches the error of the $\text {HME}_8$ for $N_{\text {sub}}=5$. The large threshold values $(\epsilon _{\textrm{C}},\epsilon _{\textrm{R}})_\textrm{l}$ yield larger errors than the $\text {HME}_{8}$, regardless of $N_\text {sub}$, as a result of the oscillation observed in Figs. 6b and c. Table 2 further shows the relative runtimes of the HME models and the A-HME, relative to the runtime of the highest-order $\text {HME}_{12}$. Apart from the A-HME simulations with the large threshold values $(\epsilon _{\textrm{C}},\epsilon _{\textrm{R}})_\textrm{l}$, the A-HME simulations yield computational speedups of up to 20 percent compared to non-adaptive HME simulations that reach similar accuracy. For this test case, the A-HME gives good results while speeding up the HME simulations.

**Table 2:** Relative error and relative runtime comparison of the$\text {HME}_{M}$,*M=2,4,… ,12*, and the A-HME, for the shock tube (52) with$\text {Kn}=0.05$. The relative errors (with respect to the DVM reference solutions) and the relative runtimes (with respect to the highest-order$\text {HME}_{12}$) are compared for different values of the minimum subdomain size parameter$N_{\text {sub}}$and for different combinations of the threshold parameters$(\epsilon _{\textrm{C}},\epsilon _{\textrm{R}})$. A-HME yields accurate results while obtaining a computational speedup, apart from large threshold values$(\epsilon _\textrm{C},\epsilon _\textrm{R})_\textrm{l}$

Model	Relative error	Relative runtime		
HME$_{2}$	3.40$\times 10^{-1}$	0.07		
HME$_{4}$	3.90$\times 10^{-2}$	0.14		
HME$_{6}$	2.56$\times 10^{-2}$	0.26		
HME$_{8}$	2.23$\times 10^{-2}$	0.43		
**HME**$_{\textbf{10}}$	**2.12**$\times {\textbf {10}}^{\mathbf{-2}}$	**0.68**		
HME$_{12}$	2.12$\times 10^{-2}$	1.00		

Model	$N_{\text {sub}}$	$(\epsilon _{\mathrm C},\epsilon _{\mathrm R})$	Relative error	Relative runtime
A-HME	20	$\boldsymbol{(\epsilon _{\textrm{C}},\epsilon _{\textrm{R}})_\textrm{s}}$	**2.12**$\times {\textbf {10}}^{\mathbf{-2}}$	**0.56**
		$(\epsilon _{\textrm{C}},\epsilon _{\textrm{R}})_\textrm{m}$	2.14$\times 10^{-2}$	0.48
		$(\epsilon _{\textrm{C}},\epsilon _{\textrm{R}})_\textrm{l}$	2.29$\times 10^{-2}$	0.48
	10	$(\epsilon _{\textrm{C}},\epsilon _{\textrm{R}})_\textrm{s}$	2.13$\times 10^{-2}$	0.55
		$(\epsilon _{\textrm{C}},\epsilon _{\textrm{R}})_\textrm{m}$	2.16$\times 10^{-2}$	0.47
		$(\epsilon _{\textrm{C}},\epsilon _{\textrm{R}})_\textrm{l}$	2.30$\times 10^{-2}$	0.47
	5	$(\epsilon _{\textrm{C}},\epsilon _{\textrm{R}})_\textrm{s}$	2.15$\times 10^{-2}$	0.54
		$(\epsilon _{\textrm{C}},\epsilon _{\textrm{R}})_\textrm{m}$	2.20$\times 10^{-2}$	0.47
		$(\epsilon _{\textrm{C}},\epsilon _{\textrm{R}})_\textrm{l}$	2.30$\times 10^{-2}$	0.47

#### Shock tube with Kn = 0.5

**Fig. 8:**
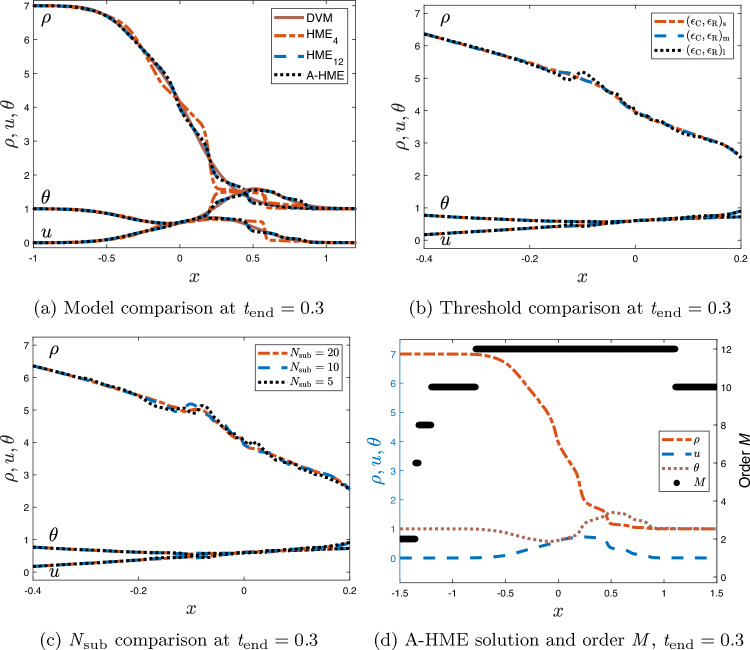
Shock tube with Kn = 0.5 and $t_{\text {end}}=0.3$. All plots show solutions for the density *ρ*, velocity *u* and temperature *θ*. (**a**): Model comparison of $\text {HME}_{4}$ (red, dash-dotted), $\text {HME}_{12}$ (blue, dashed), A-HME with minimum subdomain size $N_{\text {sub}}=10$ and with small threshold values $(\epsilon _\textrm{C},\epsilon _\textrm{R})_\textrm{s}=({1 \times 10^{-4}},{1.5 \times 10^{-4}})$ (black, dotted), and DVM reference solutions (brown, solid). (**b**): Comparison of the A-HME solutions that use minimum subdomain size $N_{\text {sub}}=10$ for different threshold values $(\epsilon _\textrm{C},\epsilon _\textrm{R})_\textrm{s}=({1 \times 10^{-4}},{1.5 \times 10^{-4}})$ (small thresholds, red dash-dotted line), $(\epsilon _\textrm{C},\epsilon _\textrm{R})_\textrm{m}=({3 \times 10^{-4}},{4.5 \times 10^{-4}})$ (moderate thresholds, blue dashed line) and $(\epsilon _\textrm{C},\epsilon _\textrm{R})_\textrm{l}=({1 \times 10^{-3}},{1.5 \times 10^{-3}})$ (large thresholds, black dotted line). (**c**): Comparison of A-HME solutions using the large threshold values $(\epsilon _\textrm{C},\epsilon _\textrm{R})_\textrm{l}$ for minimum subdomain sizes $N_\text {sub}=20$ (red, dash-dotted), $N_\text {sub}=10$ (blue, dashed), and $N_\text {sub}=5$ (black, dotted). (**d**): Final domain decomposition of the A-HME simulation with $N_\text {sub}=10$ and with threshold values $(\epsilon _\textrm{C},\epsilon _\textrm{R})_\textrm{s}$, together with the A-HME solutions of *ρ* (red, dash-dotted), *u* (blue, dashed), and *θ* (black, dotted). An oscillation is visible around *x≈ -0.1* for *ρ* in the A-HME solutions with large threshold values $(\epsilon _\textrm{C},\epsilon _\textrm{R})_\textrm{l}$ and with $N_\text {sub}=5$. The oscillation is damped for the largest minimum subdomain size $N_\text {sub}=20$

The results for large $\text {Kn}=0.5$ are shown in Fig. 8.

Figure 8a shows a comparison of the lower-order $\text {HME}_4$, the highest-order $\text {HME}_{12}$, the adaptive A-HME with minimum subdomain size $N_\text {sub}=10$ and small threshold values $(\epsilon _\textrm{C},\epsilon _\textrm{R})_\textrm{s}$, and the DVM reference solution. While the highest-order $\text {HME}_{12}$ and the adaptive A-HME yield similar results that are accurate compared to the DVM reference solution, the lower-order $\text {HME}_4$ yields poor results. Another observation in Fig. 8a is that already the error of the highest-order $\text {HME}_{12}$ with respect to the reference DVM simulations is larger compared to the results with small $\text {Kn}=0.05$ in Sect. 4.2.1. This is the result of the well-known observation that for larger Kn many moments are needed for accurate simulations because of the hyperbolic character of Grad-type moment equations (Struchtrup 2005).

Figure 8b compares the results of the A-HME with minimum subdomain size $N_\text {sub}=10$ for the different threshold values. Only for the large threshold values $(\epsilon _\textrm{C},\epsilon _\textrm{R})_\textrm{l}$, a significant oscillation is visible in the density *ρ* around *x≈ -0.1*, similar to what has been observed for $\text {Kn}=0.05$ in Sect. 4.2.1.

To investigate the effect of the minimum subdomain size parameter on this oscillation, Fig. 8c compares the A-HME results with large threshold values $(\epsilon _\textrm{C},\epsilon _\textrm{R})_\textrm{l}$ for different minimum subdomain sizes $N_\text {sub}\in \{5,10,20\}$. Interestingly, smoothing the domain decomposition by increasing the minimum subdomain size to $N_\text {sub}=20$ dampens the oscillation, as can be observed in Fig. 8c. Moreover, by reducing the minimum subdomain size to $N_\text {sub}=5$, the magnitude of the oscillation slightly increases.

Finally, the domain decomposition of the A-HME with $N_\text {sub}=10$ and small threshold values $(\epsilon _\textrm{C},\epsilon _\textrm{R})_\textrm{s}$ at $t_{\text {end}}=0.3$ is shown in Fig. 8d, which shows the order *M* in each cell together with *ρ*, *u* and *θ*. The domain decomposition assigns higher orders to regions with larger gradients of *ρ*, *u* and *θ*. In almost the entire shock region, for example, the maximum order $M_\text {max}$ is chosen. Furthermore, the orders are generally higher compared to the shock tube with small $\text {Kn}=0.05$ in Sect. 4.2.1 since there is more non-equilibrium due to the larger Kn, as has already been observed in the test case of a density shock wave colliding with smooth density wave in Sect. 4.1.

The observations in Fig. 8 are confirmed in Table 3, which gives the relative 2-norm errors (with respect to the DVM reference solutions) and the relative runtimes (with respect to the highest-order $\text {HME}_{12}$) of the different HME models and of the A-HME model with different values of the minimum subdomain size parameter $N_{\text {sub}}$ and the threshold parameters $\epsilon _\textrm{C}$ and $\epsilon _\textrm{R}$, for large $\text {Kn}=0.5$. As observed in Fig. 8a and confirmed in Table 3, the smallest threshold values $(\epsilon _{\textrm{C}},\epsilon _{\textrm{R}})_\textrm{s}$ yield accurate results, close to the error of the $\text {HME}_{12}$. Moreover, the accuracy is not affected by varying the minimum subdomain size parameter $N_\text {sub}$. This is also observed for the moderate threshold values $(\epsilon _{\textrm{C}},\epsilon _{\textrm{R}})_\textrm{m}$. The large threshold values $(\epsilon _{\textrm{C}},\epsilon _{\textrm{R}})_\textrm{l}$ yield larger errors than the small threshold $(\epsilon _{\textrm{C}},\epsilon _{\textrm{R}})_\textrm{s}$ values and the moderate threshold values $(\epsilon _{\textrm{C}},\epsilon _{\textrm{R}})_\textrm{m}$, especially in combination with the small minimum subdomain size $N_\text {sub}=5$, as already hinted at in Fig. 5d. Regarding the relative runtime, the A-HME consistently obtains computational speedups of up to 40 percent compared to non-adaptive HME simulations that reach similar accuracy.

The test cases illustrate that the A-HME method works well and leads to small errors and significant speedups if the method parameters $N_\text {sub}$, $\epsilon _{\textrm{C}}$ and $\epsilon _{\textrm{R}}$ are chosen well.

**Table 3:** Relative error and relative runtime comparison of the $\text {HME}_{M}$, *M=2,4,… ,12*, and the A-HME, for the shock tube (52) with $\text {Kn}=0.5$. The relative errors (with respect to the DVM reference solutions) and the relative runtimes (with respect to the highest-order$\text {HME}_{12}$) are compared for different values of the minimum subdomain size parameter $N_{\text {sub}}$ and for different combinations of the threshold parameters $(\epsilon _{\textrm{C}},\epsilon _{\textrm{R}})$. A-HME yields accurate results while obtaining a computational speedup

Model	Relative error	Relative runtime		
HME$_{2}$	5.48$\times 10^{-1}$	0.07		
HME$_{4}$	2.96$\times 10^{-1}$	0.15		
HME$_{6}$	2.27$\times 10^{-1}$	0.26		
HME$_{8}$	1.72$\times 10^{-1}$	0.44		
HME$_{10}$	1.41$\times 10^{-1}$	0.68		
**HME**$_{\textbf{12}}$	1.10$\times {\textbf {10}}^{\mathbf{-1}}$	**1.00**		

Model	$N_{\text {sub}}$	$(\epsilon _{\mathrm C},\epsilon _{\mathrm R})$	Relative error	Relative runtime
A-HME	20	$(\epsilon _{\textrm{C}},\epsilon _{\textrm{R}})_\textrm{s}$	1.11$\times 10^{-1}$	0.70
		$\boldsymbol{(\epsilon _{\textrm{C}},\epsilon _{\textrm{R}})_\textrm{m}}$	**1.10**$\times {\textbf {10}}^{\mathbf{-1}}$	**0.59**
		$(\epsilon _{\textrm{C}},\epsilon _{\textrm{R}})_\textrm{l}$	1.14$\times 10^{-1}$	0.58
	10	$(\epsilon _{\textrm{C}},\epsilon _{\textrm{R}})_\textrm{s}$	1.11$\times 10^{-1}$	0.68
		$(\epsilon _{\textrm{C}},\epsilon _{\textrm{R}})_\textrm{m}$	1.11$\times 10^{-1}$	0.58
		$(\epsilon _{\textrm{C}},\epsilon _{\textrm{R}})_\textrm{l}$	1.13$\times 10^{-1}$	0.57
	5	$(\epsilon _{\textrm{C}},\epsilon _{\textrm{R}})_\textrm{s}$	1.11$\times 10^{-1}$	0.68
		$(\epsilon _{\textrm{C}},\epsilon _{\textrm{R}})_\textrm{m}$	1.12$\times 10^{-1}$	0.56
		$(\epsilon _{\textrm{C}},\epsilon _{\textrm{R}})_\textrm{l}$	1.31$\times 10^{-1}$	0.55

## Conclusion

Analytical model-error estimators for the one-dimensional Hyperbolic Moment Equations (HME) were proposed and used to define a space- and time-adaptive moment model for multiscale rarefied gas microflows. First, a domain decomposition procedure was presented, in which the domain is split into subdomains modeled by different-order HME models. Second, a spatial coupling formula was discussed for coupling different-order subdomains. The decomposition is based on a numerical evaluation of the exact model difference between a higher- and a lower-order HME model. The model-error estimators are evaluated in each cell and compared with two threshold parameters for model refinement and model coarsening, while a minimum subdomain size is enforced to reduce unphysical oscillations. The coupling formula uses a path-conservative scheme with higher numerical viscosity than the one used in the interior of the subdomains.

Numerical tests of a shock tube wave colliding with a smooth density wave and of a single shock tube for different Knudsen numbers show that the analytical error estimator can capture space- and time-varying degrees of rarefaction. The results also indicate that the threshold parameters should be chosen sufficiently small to avoid unphysical oscillations, and that smoothing the domain decomposition alone is only effective in some cases. For the shock tube case, the results were compared with benchmark data from a DVM method that has been validated against experimental data in the literature. The model-adaptive method shows the ability to speed up simulations of rarefied gas flows by up to 40 percent while maintaining small errors.

Despite these promising results, the adaptive method remains limited by its one-dimensional formulation, whereas practical applications often require multi-dimensional settings. The tolerance parameters also involve a trade-off: larger tolerance values increase computational speedup by lowering the local orders, but may introduce small boundary interface oscillations, while smaller tolerance values reduce oscillations but lead to higher orders and less computational speedup. This shows that the performance of the adaptive method depends on the chosen threshold parameters, which were selected here based on preliminary numerical experiments. Since suitable threshold values are problem dependent, a systematic selection strategy remains an important topic for future work; nevertheless, the results indicate robustness over a moderate parameter range. A key advantage of the proposed methods is that they can be readily extended to multiple dimensions by applying them separately in each dimension, which is left for future research. We expect the speedup for the multidimensional case to be similar to that in the one-dimensional case, due to two main effects: On the one hand, more refinement/coarsening conditions might need to be checked as a larger number of moments are involved (e.g., in two-dimensional physical space, the models include 25 moments for a tensorized *M=4* ansatz). On the other hand, the adaptive model can also reduce this larger number more effectively than in the one-dimensional case (e.g., the two-dimensional tensorized ansatz reduces already to 16 moments for *M=3*).

Future work should therefore extend the methods to multi-dimensional configuration, consider more test cases, investigate the influence of the minimum subdomain size and threshold parameters on performance and runtime, and develop a more stable coupling of different-order subdomains to improve robustness for larger threshold values and enable greater computational speedup.

## Data Availability

The data that support the findings of this study are available from the corresponding author, R.V., upon reasonable request.

## Code availability

The code used to generate the results for the model-adaptive one-dimensional Hyperbolic Moment Equations is publicly available at: https://github.com/verbiestrik/PhD-RUG.
